# Supplementary material for: Improving outcomes for primary school children at risk of cerebral visual impairments (the CVI project): study protocol for the process evaluation of a feasibility cluster-randomised controlled trial
Source: BMJ Open. 2021 May 5;11(5):e044856. doi: 10.1136/bmjopen-2020-044856 (PMC8103382; doi:10.1136/bmjopen-2020-044856)
Supplement: Supplementary data [file bmjopen-2020-044856supp001.pdf]

Supplementary materials

A. Teacher CVI knowledge assessment survey

	0	1	2	3	4	5	6	7	8	9	10
1. I know everything about CVI	Not at all										Very much so
2. I understand how complicated the cerebral visual system is	Not at all										Very much so
3. I understand the impact of CVI in daily life	Not at all										Very much so
4. I can explain the impact of CVI in daily life to others.	Not at all										Very much so
5. I know how to adjust the environment of a child with CVI	Not at all										Very much so

B. School staff Interview Topic Guides (Control and Intervention)

CONTROL SCHOOLS TOPIC GUIDE

Introduction

- Check participant is still happy to be involved with the research
- Explain recording device and confidentiality
- Explain purpose of the interview and that participant does not have to talk about anything they feel uncomfortable with and can stop at any time for any reason

Your role

Topics to include:

- Years in role
- Involvement with SEN
- Brief School ethos overall

Experiences with the CVI Project survey collection

Topics/questions to include:

- What was it that first interested you in the study?
- How has communication been with the study team? Anything that we could have done better or made it easier for you (as a member of staff?)
- What did you think about the research agreement process?
- Data collection-how did you feel about that?
- Were the questionnaires? acceptable? Interesting? Too long? Too short?
- Randomisation experiences and feelings
- Classroom photographs

Changes during study period

Topics to include:

- Changes to provision, SEN or other
- How are changes monitored and recorded?
- Are they related to the study or not?
- Changes to policy documents -where did those changes come from? And how does that impact on your practice
- Positive, negative or unintended consequences of trial participation

INTERVENTION SCHOOLS TOPIC GUIDE

Introduction

- Check participant is still happy to be involved with the research
- Explain recording device and confidentiality
- Explain purpose of the interview and that participant does not have to talk about anything they feel uncomfortable with and can stop at any time for any reason

Your role

Topics to include:

- Years in role
- Involvement with SEN
- Brief School ethos overall

Brief Survey Questions

1. **How was the training delivered?**
2. **Who delivered the training?**
3. **Which components of the intervention pack were used?**
4. **Who received the training? How many people in each group and in total?**
5. **How many referral letters sent out to parents if any so far?**
6. **How many students/children identified as would benefit form a referral so far?**

Experiences with the CVI Project survey collection

Topics/questions to include:

- What was it that first interested you in the study?
- How has communication been with the study team? Anything that we could have done better or made it easier for you (as a member of staff?)
- What did you think about the research agreement process?
- Data collection-how did you feel about that?
- Were the questionnaires? acceptable? Interesting? Too long? Too short?
- Randomisation experiences and feelings
- Classroom photographs

Changes during study period

Topics to include:

- **The intervention pack**: initial thoughts about the content and presentation?
- **Training provided**, scope, reach, whose responsibility was it?
- **The intervention**
    - Time and impact on teachers/school/yrs 3, 4 and 5 up to July 2020; yrs 4, 5 and 6 from September 2020
    - What was engagement like from staff?
    - Use in real life, which strategies were used and for who? How did that go?
    - Changes to provision, SEN or other
    - How were changes monitored and recorded?

    - Referrals to eye service experiences, process, feedback, changes, would onsite assessments be better?
    - Changes to policy documents -where did those changes come from? Any intention to change policy in future following intervention?
- Positive, negative or unintended consequences of trial participation
- Accessing specialist provision post-covid, how and source of provision/support

C. Parent interview topic guide (Control and Intervention)

Introduction

- Check participant is still happy to be involved with the research
- Explain recording device and confidentiality
- Explain purpose of the interview and that participant does not have to talk about anything they feel uncomfortable with and can stop at any time for any reason

Your role

Topics to include:

- Describe your children
- Involvement with SEN – existing service use, referrals and stage of process.
- School ethos overall

Experiences with the CVI Project survey collection

Topics to include:

- Data collection: paper forms, returning process, any problems?
- Surveys - acceptable? Interesting? Too long? Too short?
- Randomisation experiences and feelings
- Anything not clear or troubling in surveys?

Interview part 2: Changes during study period

Topics to include:

- Control schools:
    - Changes to provision, SEN or other
    - Did you notice any changes in what is provided for your child at school?
    - Are they related to the study or not? eg classroom changes?
    - Did this impact on anything at home?
    - Positive, negative or unintended consequences of trial participation

- Intervention schools:
    - Changes to provision, SEN or other
    - Did you notice any changes in what is provided for your child at school?
    - Are they related to the study or not? eg classroom changes?
    - Did this impact on anything at home?
    - Positive, negative or unintended consequences of trial participation
    - Referrals to eye service experiences, process, feedback, changes, useful?

D. Hospital eye-service staff interview topic guide

Introduction

- Check participant is still happy to be involved with the research
- Explain recording device and confidentiality
- Explain purpose of the interview and that participant does not have to talk about anything they feel uncomfortable with and can stop at any time for any reason

Your role

Topics to include:

- Years in role
- Involvement with SEN
- Involvement with CVI –previous experience of managing children with CVI

Experiences with the CVI project referral process

Topics to include:

- How did the referral process from schools to eye unit work?
- Timing of referrals, accuracy, ability to book appointments
- Managing additional workload
- Scaling up of intervention if helpful - potential barriers/problems

Experiences with the CVI Project data collection

Topics to include:

- Training to do the visual assessments, experiences and opinions
- Patient expectations for referrals, problems identified, any diagnoses?
- Anything you would change in the research data collection (the Looking Activities)?
- Data entry using REDCap, easy to use?

Feedback to families and schools

Topics to include:

- Parent/carer reactions to appointments and feedback
- Any school responses or contact

Overall experience

Topics to include:

- Positive, negative or unintended consequences of trial participation
