# Supplementary material for: Improving outcomes for primary school children at risk of cerebral visual impairments (the CVI project): study protocol for the process evaluation of a feasibility cluster-randomised controlled trial
Source: BMJ Open. 2021 May 5;11(5):e044856. doi: 10.1136/bmjopen-2020-044856 (PMC8103382; doi:10.1136/bmjopen-2020-044856)
Supplement: Author's manuscript [file bmjopen-2020-044856.draft_revisions.pdf]

BMJ Open is committed to open peer review. As part of this commitment we make the peer review history of every article we publish publicly available.

When an article is published we post the peer reviewers' comments and the authors' responses online. We also post the versions of the paper that were used during peer review. These are the versions that the peer review comments apply to.

The versions of the paper that follow are the versions that were submitted during the peer review process. They are not the versions of record or the final published versions. They should not be cited or distributed as the published version of this manuscript.

BMJ Open is an open access journal and the full, final, typeset and author-corrected version of record of the manuscript is available on our site with no access controls, subscription charges or pay-per-view fees (http://bmjopen.bmj.com).

If you have any questions on BMJ Open's open peer review process please email info.bmjopen@bmj.com

BMJ Open

Improving outcomes for primary school children at risk of cerebral visual impairments (The CVI Project): study protocol for the process evaluation of a feasibility cluster-randomised controlled trial

Journal:	*BMJ Open*
Manuscript ID	bmjopen-2020-044856
Article Type:	Protocol
Date Submitted by the Author:	15-Sep-2020

Complete List of Authors:	Pease, Anna; University of Bristol,<br>Goodenough, Trudy; University of Bristol, Bristol Medical School<br>Sinai, Parisa; University of Bristol<br>Breheny, Katie; University of Bristol, Population Health Sciences<br>Watanabe, Rose; University of Bristol<br>Williams, Cathy; University of Bristol

Keywords:	EPIDEMIOLOGY, Developmental neurology & neurodisability < PAEDIATRICS, Paediatric ophthalmology < OPHTHALMOLOGY

SCHOLARONE™
Manuscripts

I, the Submitting Author has the right to grant and does grant on behalf of all authors of the Work (as defined in the below author licence), an exclusive licence and/or a non-exclusive licence for contributions from authors who are: i) UK Crown employees; ii) where BMJ has agreed a CC-BY licence shall apply, and/or iii) in accordance with the terms applicable for US Federal Government officers or employees acting as part of their official duties; on a worldwide, perpetual, irrevocable, royalty-free basis to BMJ Publishing Group Ltd ("BMJ") its licensees and where the relevant Journal is co-owned by BMJ to the co-owners of the Journal, to publish the Work in this journal and any other BMJ products and to exploit all rights, as set out in our licence.

The Submitting Author accepts and understands that any supply made under these terms is made by BMJ to the Submitting Author unless you are acting as an employee on behalf of your employer or a postgraduate student of an affiliated institution which is paying any applicable article publishing charge ("APC") for Open Access articles. Where the Submitting Author wishes to make the Work available on an Open Access basis (and intends to pay the relevant APC), the terms of reuse of such Open Access shall be governed by a Creative Commons licence – details of these licences and which Creative Commons licence will apply to this Work are set out in our licence referred to above.

Other than as permitted in any relevant BMJ Author's Self Archiving Policies, I confirm this Work has not been accepted for publication elsewhere, is not being considered for publication elsewhere and does not duplicate material already published. I confirm all authors consent to publication of this Work and authorise the granting of this licence.

For peer review only

Improving outcomes for primary school children at risk of cerebral visual impairments (The CVI Project): study protocol for the process evaluation of a feasibility cluster-randomised controlled trial

Anna Susan Pease[1], Trudy Goodenough[1], Parisa Sinai[1], Katie Breheny[2], Rose Watanabe[1], Cathy Williams[1]

[1] Centre for Academic Child Health, Bristol Medical School, University of Bristol, 1-5 Whiteladies Road, BS8 1NU, Bristol, UK
Email: a.pease@bristol.ac.uk
[2] Population Health Sciences, Bristol Medical School, University of Bristol, Bristol, UK

Word count: 3094 (limit 4000)

Corresponding Author: C Williams, 1-5 Whiteladies Road, Bristol BS8 1NU
Cathy.Williams@bristol.ac.uk

For peer review only

Abstract

Introduction

Brain related visual impairments, also known as Cerebral Visual Impairment or CVI are related to damage or poor function in the vision related areas of the brain. Recognising CVI, however defined, in children is difficult and many children with CVI may not get the help they need. The CVI Project is a programme of work relating to CVI and includes the development of a complex intervention to share knowledge with teachers so that they can make both targeted and universal changes to support children with CVIs. A feasibility study for a cluster randomised controlled trial to evaluate this intervention is underway. This paper describes the protocol for an accompanying Process Evaluation (PE) to explore how the intervention is implemented and provide context for the interpretation of the feasibility trial outcomes.

Methods and analysis

A logic model has been developed to guide data collection. Both qualitative and quantitative data will be collected to assess the feasibility and acceptability of the intervention, the study design, and explore how any changes that occur are brought about. Interviews with key primary school staff and parents will investigate responses to the intervention and trial processes. Surveys will collect data on intervention implementation and knowledge of CVI. Photographs of classroom walls will measure changes to visual clutter and document analysis will look for changes to school SEND policies. Interviews with orthoptists will investigate the referral process and responses to the functional visual assessments.

Ethics and dissemination

Ethical approval was granted by the University of Bristol Faculty of Health Sciences Ethics Committee. Findings will contribute to the development of a full-scale cluster RCT to assess the effectiveness of the intervention with adequate statistical power. The results will also support the refinement of the intervention, and its underlying theory.

Keywords: Cerebral Visual Impairment, Complex intervention, feasibility trial, process evaluation, child.

Strengths and limitations of this study

- **Intervention developed with input from professionals and families**
- **Novel intervention to improve awareness of CVI and strategies to help**
- **Process evaluation designed to inform future full-scale trial, if warranted**
- **Study designed using programme logic to inform data collection timelines and methods**
- **Ethical approval re-established following COVD-19 related pause in the study**

For peer review only

INTRODUCTION

Damage or poor function in the brain areas related to vision leads to brain-related visual impairments, also known collectively as cerebral visual impairment (CVI) or sometimes in the plural (CVIs) (1, 2). CVIs in children can manifest in many ways; for example, being unable to find things in a cluttered page; bumping into people; being unable to copy from the class whiteboard to their own workbooks or difficulties controlling their eye position effectively to keep focused on a task (3). It has been reported that vision problems may be misinterpreted by teachers as behavioural or other difficulties (4) and so children with CVI/s may not get the help they need. Concern has been raised that missing a diagnosis of CVI can affect mental health(5, 6), and research from a UK birth cohort suggested that children with CVI may experience academic difficulties(7). Research into strategies to support children with CVI has suggested that providing a less cluttered visual environment may help (8, 9).

In preparation for this study, discussions with two parent advisory groups, and with an advisory group comprising local health and education professionals, have reinforced accounts that simple adaptations of the child's visual world can be very helpful. They emphasize children's improved sense of well-being when they can achieve more and are not chastised for behaviour resulting from their CVIs. They have told us that access to help is uneven between and within different areas of the country, and therefore is inequitable, and that few teachers, SENCo (Special Educational Needs Co-ordinator) or Learning Support Assistants (LSAs) know about CVI. In general, only children with reduced visual acuity have access to specialist vision support teachers (QTVIs) and so many children with CVI but good visual acuity, are ineligible for their help. A prevalence study conducted by our group has indicated that there are likely to be at least 3% of children with CVI-related vision problems in primary schools in England and that these are largely but not exclusively within the subset of children already identified as needing extra educational help (paper under review).

Aim of the Process Evaluation

The aim of the CVI Project process evaluation is to understand both the functioning of the trial processes and the intervention itself. This understanding is crucial to the development and decision to proceed to the full trial.

METHODS AND ANALYSIS

The CVI Project Intervention

We have developed an intervention which seeks to share knowledge with teachers so that they can make both universal and targeted changes within their school, to better support children with CVIs and potentially other children too(9).

The development of the intervention is based on recent guidance, including a review of the published literature, using an iterative process of development and refining, involving key stakeholders, developing an underlying theory that takes into account the context and possible real world implementation issues (10). The key elements of the intervention and the hypothesised outcomes are summarised in a Logic model - see Figure 1.

We hypothesize that this multilevel approach involving both schools and the hospital service, will improve educational and wellbeing-related outcomes for any child with CVI and potentially for their peers as well. We have therefore devised an information-sharing pack to give schools this information. This includes an overview and explanations of the condition and gives suggestions as to how to make the school more "vision friendly". Central to this is to use universal approaches

For peer review only

wherever possible - for instance decluttering walls and classrooms, using larger font and clear layouts with worksheets etc. Additional targeted strategies, for use with at-risk children, will also be given, with explanations about who might benefit from these, how they are intended to work and their potential impact. The information included in the intervention pack for teachers is collated from several centres of excellence that have developed materials about CVIs, including Bartiméus in the Netherlands (11) and the international TeachCVI initiative (12). The intervention also includes sharing information with the local paediatric ophthalmology clinics about the presentations of and support for children with CVI, so that they are happy to accept referrals of at-risk children. The study team will liaise with a Paediatric Ophthalmologist in the ophthalmology clinic so that they are prepared to accept referrals for the management of children suspected of having CVIs. Intervention schools will be able to refer a maximum 5% of children to the paediatric ophthalmology clinic for a functional visual CVI assessment. A printout of the CVI assessment will be available to the family and school with strategies where applicable.

A logic model showing proposed mechanisms of change, impact and outcomes is shown in Figure 1. This model has been refined throughout our intervention development with parents and professionals, and includes consideration of unintended negative consequences of the intervention, or 'dark logic', as proposed by Bonell and colleagues (13).

Figure 1: CVI Project Intervention Logic Model

The CVI Project Feasibility Trial

The intervention and trial processes are being tested in a cluster randomised feasibility trial, with nested process and health economic evaluations. The main aim of this trial is to assess the feasibility and acceptability of our intervention and trial methods, to inform the development of a future adequately-powered cluster-randomised controlled trial, if warranted (14). The full protocol for the feasibility trial is presented separately and is summarised here, to give context for the Process Evaluation, which is described in full below.

The feasibility trial involves mainstream primary schools in three regions of the UK: Gloucester, Hampshire and Somerset. Baseline and follow up data will be collected on students in years 3-5 attending participating schools. In this trial the primary outcome will be assessed in all children who are getting extra help, rather than only in children with confirmed CVI. This is because our previous study suggested most children with CVI are within the group getting extra help and these children are easily identifiable by schools (whereas children with confirmed CVI are not). However, we also know some children with CVI are not getting extra help and we hypothesize many children might benefit from some aspects of the intervention e.g. decluttering. We will therefore collect data for all children in Y3-5; the Primary outcome will be assessed in the children getting extra help, but we will include all children in a secondary outcome analysis.

After baseline data are collected, a statistician separate from the study will randomise the participating schools, with two stratification variables to maintain similar sample sizes in each arm (15) 1) 15% or less of children having extra help or more than 15% of children and 2) size of school (3+ classes per year vs 2 or fewer classes per year, in years 3-5). Schools will be told immediately which group they are allocated to. Schools allocated to the control group will be asked to carry on as normal for the school year.

For peer review only

Some of the outcomes of the Feasibility study will be assessed using data collected during the study in a process evaluation. The process evaluation will use a mixture of quantitative and qualitative methods for data collection. Complex interventions are described as those which contain several interacting components and may also relate to the implementation of the intervention and its interaction with its context (16). Process evaluations embedded within complex interventions aim to understand the functioning of an intervention by examining implementation, mechanisms of impact and contextual factors (17). There are several frameworks which combine these aspects of process evaluation within feasibility of complex interventions and RCTs; for this study our framework is based on guidance from the Medical Research Council (MRC) (16). We have used this guidance to develop the aims and conceptual framework for the PE. These are described below.

The seven domains to be evaluated as recommended by MRC guidance are as follows:

1. *Implementation of the intervention*

This domain aims to discover what is implemented in practice. We will consider how the CVI Project Intervention Training package is used in each school. We will assess the quality (fidelity) of the intervention and how much of the intervention (dose) was delivered and to whom (reach). We will also determine if there have been any adaptations to the intervention and the reason or purpose of these adaptations. Consideration will also be given to the core components of the intervention, and the barriers and facilitators which influence how individual components of the intervention are selected and delivered.

2. *Mechanism of Impact/change*

The process evaluation will consider how the intervention as delivered by schools, worked. Research questions will provide information and insights about how the participants, schools and parents responded to and interacted with the intervention. Questions will examine changes in the understanding and knowledge of staff about CVI and how this interacted with their implementation of the intervention, along with exploring the barriers and facilitators they experienced to implementation. Questions will also consider if there were any unintended or unexpected consequences or pathways of the intervention.

3. *Programme Differentiation and Usual Practice*

The research questions in this domain will examine how the intervention differed from usual practice in the intervention schools. Contamination from intervention to control schools will also be assessed and exploratory questions will discover how and why this occurred and the outcome for control schools.

4. *Acceptability of the intervention*

This domain will consider the interactions of participants, both school staff and parents with the intervention. The effects of acceptability on implementation of the intervention will be explored. We will consider if acceptability changes across the school year, and how the responses and interactions of staff and parents with the intervention might change the way the intervention is implemented.

5. *Sustainability*

This domain will consider whether the intervention could become part of normal practice after the feasibility trial was complete. We will assess the fidelity, dose and reach of the intervention in is sustained form. Where it has not been sustained, or there are no plans to continue with the changes after the trial, we will explore why this is. Barriers and facilitators to sustainability will also be explored.

For peer review only

6. Context

Contextual factors may influence the effectiveness of an intervention both directly and indirectly. These are external factors which can impede or strengthen its implementation or effects (MRC). We will consider how the paediatric ophthalmology clinics respond and interact with the intervention, including the referral pathway from schools to HES. In addition, this domain will consider the wider contextual factors which could affect the implementation of the intervention.

7. Trial Process

Understanding the participants responses to and interactions with the trial processes will allow an assessment of how the trial methods might require adjustment or re-design for further work. Understanding the responses of the participants will also provide information about recruitment and continued participation in the trial by both schools, teachers and parents.

Design

The CVI Project is a feasibility cluster randomised study with nested process evaluation and health economic assessment. The process evaluation will use both quantitative and qualitative data sources in order to answer our research questions. We anticipate that all schools will contribute data to the process evaluation, allowing for different experiences with the study between intervention and control arms.

Data sources

We have summarised the research domains, research questions and methods of evaluation in Table 1.

For peer review only

Table 1: Process evaluation research domains and methods of evaluation

Domain	Research questions<br><br>***Process evaluation framework mapping***	Informant/source of data	Methods	Timing of data collection	Analysis
**Implementation of intervention (the information pack)**<br><br>**Intervention Schools n=4**	Which CVI Project information pack components were delivered?<br><br>*Fidelity, dose, reach*	SENCO/head teacher n=4	Brief survey question	Post-intervention	Data recorded and tabulated
	Which resources were used?<br><br>How were these resources used?<br><br>*Fidelity*	SENCO/head teacher n=4	Brief survey questions<br><br>Semi-structured interview	Post-intervention	Data recorded and tabulated<br><br>Thematic analysis
	How was the training delivered? For example: training day, short sessions.<br><br>*Fidelity, adaptation*	SENCO/head teacher n=4	Brief survey questions	Post-intervention	Data recorded and tabulated
	Who received the training?<br><br>*Reach*	SENCO/head teacher n=4	Brief survey questions	Post-intervention	Data recorded and tabulated
	Were adaptations made to resources? How did this affect implementation?<br><br>*Adaptation, Fidelity*	SENCO/head teacher n=4	Semi-structured Interview	Post-intervention	Thematic analysis
	What were the barriers and facilitators to delivering the training?<br><br>*Mediators*	SENCO/head teacher n=4	Semi-structured Interview	Post-intervention	Thematic analysis
**Mechanisms of change of CVI Project intervention**<br><br>**Intervention Schools**	What was the response of staff to the training?<br><br>*Participant responses to and interactions with the CVI Project Intervention*	SENCOs/head teacher classroom teachers, LSA years 3-5 n=TBC	Semi-structured Interview	Post-intervention	Thematic Analysis

For peer review only

**n=4**	How did staff knowledge about CVI change in response to training/intervention?<br><br>*Participant responses, reach*	SENCO/head teacher classroom teachers,<br>LSAs years 3-5<br>n=TBC	Bartiméus Centre (Netherlands)<br>Teacher CVI Knowledge Assessment Survey	Brief online survey pre randomisation follow up data collection.	Quantitative analysis
	What were staff and parents' responses to the implementation of the CVI Project intervention?<br><br>*Participant responses to and interactions with the CVI Project Intervention*	SENCO/head teacher classroom teachers, LSA years 3-5<br>n=TBC	Semi-structured Interview	Post- intervention	Thematic analysis
	What changes to school environment took place? E.g. Whole school/class/individual child changes<br><br>*Fidelity, adaptations, and reach*<br>*Context of implementation of intervention*	SENCO/head teacher, classroom teachers, years 3-5<br>n=23 classrooms<br><br>SENCO/head teacher n=4	Photographs taken of study designated viewpoints of each wall in years 3-5 classrooms<br><br>Semi-structured interviews	Pre and post randomisation<br>Pre-follow up data collection<br><br>Post follow up data collection	Quantitative analysis using validated University of Bristol 'clutter' measure<br><br>Thematic analysis
	Were there any unintended/unexpected consequences from the intervention? What were they? How could they be mitigated (if necessary)?<br><br>*Unintended/unexpected pathways or consequences*	SENCO/head teacher, classroom teachers, LSAs years 3-5; parents<br>n=TBC	Semi-structured Interview	Post-intervention	Thematic analysis
	What were the barriers and facilitators for the implementation of the CVI Project intervention?<br><br>*Participant responses to and interactions with the CVI Project Intervention*	SENCO/head teacher, classroom teachers, LSAs years 3-5; parents<br>n=TBC	Semi-structured Interview	Post-intervention	Thematic analysis
	What was the response of staff to the training?<br><br>*Participant responses to and interactions with the CVI Project Intervention*	SENCOs/head teacher, classroom teachers, LSA years 3-5<br>n=TBC	Semi-structured Interview	Post-intervention	Thematic Analysis

For peer review only

**CVI Project intervention differentiation and usual practice**<br><br>**Intervention schools n=4**<br><br>**Control Schools n=3**	Was any contamination detected from intervention to control schools?<br><br>Were there any changes in school SEND documentation over the course of the study that might indicate contamination?<br><br>*Contamination*	SENCO/head teacher n=7 (all schools)<br><br>Head teacher/SENCO and classroom teachers, years 3-5 n=35 classes (control and intervention schools)<br><br>SEND Policy documents in all schools n=7	Semi-structured Interview<br><br>Photographs of study designated viewpoints of each wall in years 3-5 classrooms<br><br>Structured review of schools' SEND documentation	Follow up data collection<br><br>Pre-randomisation and at follow up data collection<br><br>Pre-follow up data collection	Thematic analysis<br><br>Validated University of Bristol 'clutter' measure Quantitative analysis<br><br>Content analysis
	What was different about the CVI Project Intervention when compared to usual practice within the school?<br><br>*Differentiation, mediators to implementation of the intervention*	Head teacher/SENCO and classroom teachers, years 3-5 n=TBC	Semi-structured Interview	Follow up data collection	Thematic analysis
**Acceptability of the Intervention**	Was the CVI project intervention acceptable to school staff and parents?<br><br>*Acceptability: participants' responses and interactions with the intervention*	Intervention schools: SENCO/head teacher, classroom teacher/LSA years 3-5, parents n=TBC	Semi-structured Interview	Post Follow-up	Thematic analysis
	What were the experiences of staff and parents of taking part in the CVI project intervention?<br><br>*Participant interactions with the CVI Project Intervention*	Intervention schools: SENCO/head teacher, classroom teachers, LSAs years 3-5; parents n=TBC	Semi-structured Interview	Post Follow-up	Thematic analysis
**Sustainability of the intervention**	Did schools/were schools intending to continue with intervention after the trial period?<br><br>*Sustainability, participants' responses to the intervention, fidelity, quality of sustained use.*	SENCO/head teacher<br><br>n=4	Semi-structured Interview	Post Follow-up	Thematic analysis

For peer review only

	How was the intervention integrated into usual practice?<br><br>Was intervention integrated into SEND policies?<br><br>*Mechanisms of sustainability of intervention*	SENCO/head teachers, classroom teachers, years 3-5<br>n=TBC<br><br>SEND policy documents from intervention schools n=4	Semi-structured Interview<br><br>Structured review of SEND policy documents	Post Follow-up<br><br>Pre-randomisation<br>Post Follow-up	Thematic analysis<br><br>Content analysis
	What factors made it more/less likely to be sustained/adopted into usual practice?<br><br>*Barriers and facilitators/mediators to sustainability of the intervention*	SENCO/head teacher<br><br>n=4	Semi-structured Interview	Post Follow-up	Thematic analysis
**Context**<br><br>**Intervention Schools n=4**	What were the experiences of paediatric ophthalmology clinics and orthoptists?<br><br>*Participants responses to the intervention*<br>*Barriers and facilitators to participation in the CVI Project feasibility RCT*	Head orthoptist<br>n=3	Semi-structured Interview	Post Follow-up	Thematic analysis
	How did the referral process from schools to paediatric ophthalmology clinics work?<br><br>*Fidelity, adaptations, reach, barriers and facilitators*<br>*Contamination, unintended consequences*	Paediatric ophthalmology clinics<br>n=3<br>SENCO/head teacher<br>n=4	Semi-structured Interview	Post Follow-up	Thematic analysis
	How many children were given study referral letters for their parents?<br>Who were they referred to?<br><br>What were the characteristics of the children who attended the paediatric ophthalmology clinic with a study referral letter?<br><br>*Fidelity, dose, reach*	SENCO/head teacher<br>n=4<br><br>Paediatric ophthalmology clinics<br>n=3	Records kept at each school<br>Schools collate data<br><br>CVI-vision assessment Conducted by orthoptists	Throughout trial	Count and record<br><br>Recorded on University REDCap database accessed via paediatric ophthalmology clinics.

For peer review only

	How did contextual factors affect intervention implementation? (e.g. funding pressures; reorganisation; academisation, Ofsted, SATS)<br><br>*Implementation and mechanisms of impact*	SENCO/head teacher<br>n=4	Semi-structured Interview	Pre-follow up data collection	Thematic analysis
**Trial Process**<br><br>**Intervention and Control Schools n=7**	What did schools think about the recruitment process?<br><br>*Trial methods, acceptability to participants. Participant responses to trial methods Barriers and facilitators*	Head teachers/SENCOs in control and intervention schools<br><br>n=7	Semi-structured Interview	Post baseline data collection	Thematic analysis
	What was the initial recruitment rate?<br><br>*Recruitment data*	Demographic data from publicly available government schools data base	Extraction of demographic data	Post recruitment	Descriptive data summaries
	What was the dropout rate of schools from the research? What reasons were given?<br><br>*Participant responses and interactions with the trial processes*	Recorded as part of trial routine data collection	Quantitative data with some free text/coded responses for reasons	Ongoing throughout study period	Count and record
	How many parents withdrew their children from the study? What reasons were given?<br><br>*Participant responses and interactions with the trial processes*	Recorded as part of trial routine data collection	Quantitative data with some free text/coded responses for reasons	Ongoing throughout study period	Count and record
	How did schools communicate with parents about trial?<br>*Mechanisms of impact, fidelity, reach*	Examination of school documents/websites/newsletters	Structured review of documents	Ongoing throughout study period	Content analysis
	Acceptability of trial processes: how did schools respond to randomisation? Including the arm of the trial that they were allocated to?<br><br>*Acceptability of trial processes, interactions of participants with trial processes*	SENCO/head teacher in intervention and control schools<br><br>n=7	Semi-structured interview	Post randomisation	Thematic analysis

For peer review only

Surveys

Each intervention school will complete a brief school-level survey at baseline, conducted at the beginning of the qualitative interview. The survey includes 6 questions about the implementation of the intervention pack including how it was delivered, who delivered it, which components of the intervention pack have been used, who received the training, how many referral letters have been sent out and how many students have been identified for referral. Follow up questions will include numbers who have received the intervention, which components have been used, numbers of children identified and referred.

Teacher knowledge of CVI will be assessed before randomisation and at follow up, using the Bartiméus Centre (Netherlands) Teacher CVI Knowledge Assessment Survey (18). All teachers for years 3-5 in all schools will complete these. The survey is comprised of 5 statements about knowledge of CVI (e.g. 'I understand the impact of CVI in daily life') and respondents are asked to rate whether they feel each statement is true for them on a scale of 0-10.

Qualitative interviews with school representative (all schools)

One key representative from each school (usually the SENCO or head teacher) will take part in a semi-structured interview at baseline following randomisation, (after the intervention has been delivered in intervention schools) and at follow up. The initial interview will cover school ethos, experiences with data collection, trial processes (including randomisation) and any changes during the study period. Additional topics for intervention schools will include responses to the intervention pack, resources implemented, feedback from staff, monitoring and recording of changes.

Qualitative interviews with school staff (intervention schools only)

School staff will be invited to take part in a semi-structured interview post intervention implementation, to share their experiences of the CVI Project intervention. The interviews will explore reactions to the intervention materials, how they have been used, views and opinions on their use and usefulness, changes to provision, implementation of both universal and targeted strategies and any feedback on the data collection methods used in the trial. Interviews with staff will take place as the intervention is being used at the school.

Qualitative interviews with parents (all schools)

Parents who returned 'consent to contact' forms will be contacted and invited to take part in a semi-structured interview about their experiences with the study. Interviews will explore their experiences with the data collection, surveys and any thoughts they had about randomisation. Interviews will also cover any changes during the study period and what these can be attributed to, and any referral processes and experiences with the paediatric ophthalmology clinics. Any positive, negative or unintended consequences will be discussed.

Qualitative interviews with paediatric ophthalmology clinic staff

Paediatric ophthalmology clinic staff with involvement in the study will be invited to take part in a semi-structured interview to explore their experiences with referrals from schools. The topics covered in these interviews will include the referral process, characteristics of children who are referred, communication between school, family and hospital unit.

Classroom Clutter (all schools)

For peer review only

We will ask schools to take photographs of each wall in the classrooms used by years 3-5 in each school, pre-randomisation and again at follow up. Photographs of the classroom setting will be analysed using a clutter rating scale, developed at baseline with independent blind ratings by an academic not connected with the study, but with expertise in this area (19). An objective measure of classroom visual clutter will be used in order to measure changes in levels of clutter type and amount at baseline and follow up.

School document analysis

We will investigate whether there any changes in school SEND documentation over the course of the study that might indicate contamination by conducting a structured review of all school's SEND policy documentation pre-randomisation and at follow up. They will be studied for any changes made during the study period. Changes found will be included as topics for discussion with SENCo and staff interviews.

Analysis of qualitative data

With consent, the semi-structured interviews will be audio recorded and then transcribed verbatim, anonymised, checked for accuracy and imported into NVivo12(20). Thematic analysis using a continuous comparative method will be used to identify recurrent ideas and patterns within the data (21). Transcripts will be independently coded by two researchers to enhance rigour by cross checking the coding strategy and early development of themes. During analysis, themes will be discussed among the team until a final consensus on the nature and phrasing of key themes and subthemes is reached. Anonymised quotes will be presented to illustrate the main themes.

Analysis of quantitative data

Quantitative survey data will be presented as counts and percentages, as appropriate. String variables will be coded into categories. The CVI knowledge survey will be scored and comparisons made between baseline and follow up. Survey data will be compared between intervention and control schools, but this will be exploratory and will be reported descriptively as the study is not powered to detect an effect size (14).

Photographs of the classroom setting will be analysed using a clutter rating scale, developed at baseline with independent blind ratings by an academic not connected with the study, but with expertise in this area.

School policy documents will be subject to content analysis by the study team, using a data extraction table to identify any differences between schools at the beginning and end of the study period.

ETHICS and HRA

Ethical approval for the study was obtained from the University of Bristol Faculty of Health Sciences Ethics Committee (FREC Ref: 89144). The study is registered on the International Standard Randomised Controlled Trial Number registry (registration number: ISRCTN13762177). Approval from the Health Research Authority (HRA- Ref 19/HRA/6124) has been obtained.

Full details of consent procedures are described in our feasibility trial protocol paper (in preparation), but in brief: Head teachers agree to participate on behalf of the children and staff in his/her school, information sheets will be provided to school staff and parents, parents will be able to withdraw their child's data by completing a non-agreement form. Verbal agreement for child completed measures will also be sought and questionnaires will not be completed by any child who

For peer review only

does not wish to do so. A link to the full Privacy notice online (22) will be provided to all parents and school staff.

DISCUSSION

We have presented a protocol for a detailed process evaluation of a complex information sharing intervention to improve the mental well-being of primary school children with CVI. Development of an intervention to support children with CVI has been requested by parents (23), and we have developed it with their input. This feasibility study will assess whether and how a future trial should establish effectiveness of the intervention with feedback from this process evaluation. We have addressed key domains as per MRC guidance, and have developed our process evaluation research questions around these aims. Data gathered will help us to interpret the findings from the trial, but importantly, will help to refine the design, content and delivery of the intervention. We have also included data to improve trial processes and communication between schools and the research team in a future full-scale trial. Given that this is a process evaluation for a feasibility trial, we will not be powered to interpret the primary outcome differences between intervention and control, and the data gathered as part of the process evaluation will be limited in explaining any differences in the main feasibility study outcomes.

TRIAL STATUS

Favourable opinion by the University of Bristol faculty ethics committee was obtained on 12th August 2019 (Ref: 89144). We completed recruitment and baseline data collection from participating schools in all areas. We have recruited 7 schools to the feasibility trial and completed baseline trial data collection in February 2020. Four schools have been allocated to the intervention arm and three to control. Due to the COVID-19 global pandemic and school closures, we paused the study from March - September 2020. We have since obtained ethical approval for minor amendments relating to adjustments for a 'COVID-secure' restart to the study on September 1st 2020, with an extension to the study period until January 31st 2021 We will describe all COVID related changes to the study in our final results paper, as these relate to the findings of our feasibility study.

FUNDING AND SPONSORSHIP

University of Bristol provides Sponsorship for this study. The study is funded by the National Institute for Health Research (NIHR) Senior Research Fellowship ref: SRF-2015-008

Author contributions

CW led the development of the research including the design of the intervention and trial methods, with support from AP, TG and PS. RW provided data management and systems support. PS provided governance support. AP and TG led on the design of the process evaluation and associated research methodology. DG provided statistical support and randomisation to trial arms. KB led the development of the health economics aspect of the process evaluation. All authors contributed to the final manuscript providing comments and text for the final submitted draft.

Acknowledgements

We are grateful to Ms Mai Baquedano for her support with online consent and questionnaires, via the REDCap system. We are also grateful to our family and professionals advisory group members who provided valuable insight into recruitment and data collection strategies, and input into the intervention delivery.

For peer review only

REFERENCES

1. Sakki HEA, Dale NJ, Sargent J, Perez-Roche T, Bowman R. Is there consensus in defining childhood cerebral visual impairment? A systematic review of terminology and definitions. The British journal of ophthalmology. 2018;102(4):424-32.
2. Dutton GN. Cognitive vision, its disorders and differential diagnosis in adults and children: knowing where and what things are. Eye (London, England). 2003;17(3):289-304.
3. Dutton GN, Jacobson LK. Cerebral visual impairment in children. Seminars in neonatology : SN. 2001;6(6):477-85.
4. Swift S, Davidson R, Weems L. Cortical Visual Impairment: Presentation Intervention and Prognosis in Educational Settings. TEACHING Exceptional Children PLus. 2008;4(5):1-14.
5. Lueck A, Dutton G. Vision and the Brain: AFB Press; 2015.
6. Murphy M, Fonagy P. Chief Medical Officers Report 2012. Chapter 10. Mental health problems in children and young people. In: Health UgDo, editor.: UK Government; 2012.
7. Williams C, Northstone K, Sabates R, Feinstein L, Emond A, Dutton GN. Visual perceptual difficulties and under-achievement at school in a large community-based sample of children. PLoS One. 2011;6(3):e14772.
8. McDowell N, Budd JJJoVI, Blindness. The perspectives of teachers and paraeducators on the relationship between classroom clutter and learning experiences for students with cerebral visual impairment. 2018;112(3):248-60.
9. Fisher AV, Godwin KE, Seltman H. Visual environment, attention allocation, and learning in young children: when too much of a good thing may be bad. Psychol Sci. 2014;25(7):1362-70.
10. O'Cathain A, Croot L, Duncan E, Rousseau N, Sworn K, Turner KM, et al. Guidance on how to develop complex interventions to improve health and healthcare. 2019;9(8):e029954.
11. Landis JR, Koch GG. The measurement of observer agreement for categorical data. Biometrics. 1977;33(1):159-74.
12. B&H The National Institute for the Blind Visually Impaired and Deafblind in Iceland. TeachCVI [Available from: https://www.teachcvi.net/.
13. Bonell C, Jamal F, Melendez-Torres G, Cummins SJJECH. 'Dark logic': theorising the harmful consequences of public health interventions. 2015;69(1):95-8.
14. Eldridge SM, Lancaster GA, Campbell MJ, Thabane L, Hopewell S, Coleman CL, et al. Defining feasibility and pilot studies in preparation for randomised controlled trials: development of a conceptual framework. PloS one. 2016;11(3):e0150205.
15. Treasure T, MacRae KD. Minimisation: the platinum standard for trials?: randomisation doesn't guarantee similarity of groups; minimisation does. British Medical Journal Publishing Group; 1998.
16. Moore G AS, Barker M, Bond L, Bonell C, Hardeman W, Moore L, O'Cathain A, Tinati T, Wight D, Baird J. Process evaluation of complex interventions: Medical Research Council guidance. MRC Population Health Science Research Network, London, 2014 Process evaluation of complex interventions: Medical Research Council guidance. London: MRC Population Health Science Research Network; 2014.
17. Grant A, Treweek S, Dreischulte T, Foy R, Guthrie BJT. Process evaluations for cluster-randomised trials of complex interventions: a proposed framework for design and reporting. 2013;14(1):15.
18. Pilon-Kamsteeg F, Dekker-Pap MJ, de Wit GC, van Genderen MMJBJoVI. CVI Experience toolbox: Simulation of visual processing difficulties. 2019;37(3):248-57.

For peer review only

19. Xiao F, Cuthill IC. Background complexity and the detectability of camouflaged targets by birds and humans. Proceedings of the Royal Society B: Biological Sciences. 2016;283(1838):20161527.
20. Ltd. QIP. NVivo (Version 12) 2018 [Available from: https://www.qsrinternational.com/nvivo-qualitative-data-analysis-software/home.
21. Braun V, Clarke V. Thematic analysis. 2012.
22. The CVI Project Feasibility Study Data Security and Privacy Notice [Available from: http://www.thecviproject.co.uk/privacy/.
23. Jackel B, Wilson M, Hartmann EJJoVI, Blindness. A survey of parents of children with cortical or cerebral visual impairment. 2010;104(10):613-23.

For peer review only

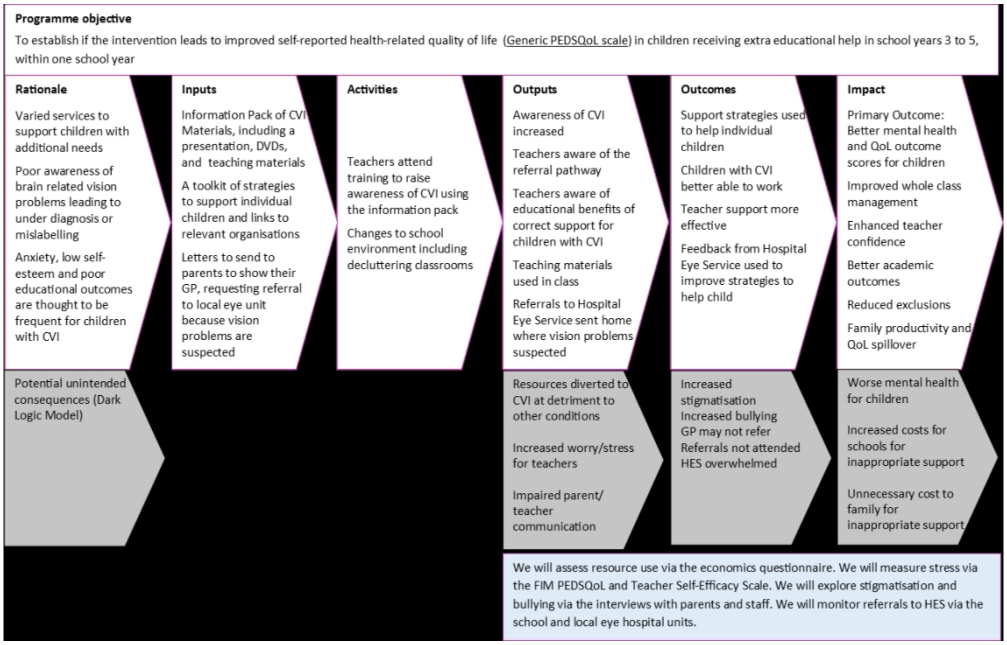
Programme objective
To establish if the intervention leads to improved self-reported health-related quality of life (Generic PEDSQoL scale) in children receiving extra educational help in school years 3 to 5, within one school year
Rationale
Varied services to support children with additional needs
Poor awareness of brain related vision problems leading to under diagnosis or mislabelling
Anxiety, low self-esteem and poor educational outcomes are thought to be frequent for children with CVI
Inputs
Information Pack of CVI Materials, including a presentation, DVDs, and teaching materials
A toolkit of strategies to support individual children and links to relevant organisations
Letters to send to parents to show their GP, requesting referral to local eye unit because vision problems are suspected
Activities
Teachers attend training to raise awareness of CVI using the information pack
Changes to school environment including decluttering classrooms
Outputs
Awareness of CVI increased
Teachers aware of the referral pathway
Teachers aware of educational benefits of correct support for children with CVI
Teaching materials used in class
Referrals to Hospital Eye Service sent home where vision problems suspected
Outcomes
Support strategies used to help individual children
Children with CVI better able to work
Teacher support more effective
Feedback from Hospital Eye Service used to improve strategies to help child
Impact
Primary Outcome: Better mental health and QoL outcome scores for children
Improved whole class management
Enhanced teacher confidence
Better academic outcomes
Reduced exclusions
Family productivity and QoL spillover
Potential unintended consequences (Dark Logic Model)
Resources diverted to CVI at detriment to other conditions
Increased worry/stress for teachers
Impaired parent/ teacher communication
Increased stigmatisation
Increased bullying
GP may not refer
Referrals not attended
HES overwhelmed
Worse mental health for children
Increased costs for schools for inappropriate support
Unnecessary cost to family for inappropriate support
We will assess resource use via the economics questionnaire. We will measure stress via the FIM PEDSQoL and Teacher Self-Efficacy Scale. We will explore stigmatisation and bullying via the interviews with parents and staff. We will monitor referrals to HES via the school and local eye hospital units.

Improving outcomes for primary school children at risk of cerebral visual impairments (The CVI Project): study protocol for the process evaluation of a feasibility cluster-randomised controlled trial

Journal:	*BMJ Open*
Manuscript ID	bmjopen-2020-044856.R1
Article Type:	Protocol
Date Submitted by the Author:	19-Jan-2021
Complete List of Authors:	Pease, Anna; University of Bristol, Bristol Medical School<br>Goodenough, Trudy; University of Bristol, Bristol Medical School<br>Sinai, Parisa; University of Bristol, Bristol Medical School<br>Breheny, Katie; University of Bristol, Bristol Medical School<br>Watanabe, Rose; University of Bristol, Bristol Medical School<br>Williams, Cathy; University of Bristol, Bristol Medical School
**Primary Subject Heading**:	Ophthalmology
Secondary Subject Heading:	Paediatrics, Research methods
Keywords:	EPIDEMIOLOGY, Developmental neurology & neurodisability < PAEDIATRICS, Paediatric ophthalmology < OPHTHALMOLOGY

SCHOLARONE™
Manuscripts

I, the Submitting Author has the right to grant and does grant on behalf of all authors of the Work (as defined in the below author licence), an exclusive licence and/or a non-exclusive licence for contributions from authors who are: i) UK Crown employees; ii) where BMJ has agreed a CC-BY licence shall apply, and/or iii) in accordance with the terms applicable for US Federal Government officers or employees acting as part of their official duties; on a worldwide, perpetual, irrevocable, royalty-free basis to BMJ Publishing Group Ltd ("BMJ") its licensees and where the relevant Journal is co-owned by BMJ to the co-owners of the Journal, to publish the Work in this journal and any other BMJ products and to exploit all rights, as set out in our licence.

The Submitting Author accepts and understands that any supply made under these terms is made by BMJ to the Submitting Author unless you are acting as an employee on behalf of your employer or a postgraduate student of an affiliated institution which is paying any applicable article publishing charge ("APC") for Open Access articles. Where the Submitting Author wishes to make the Work available on an Open Access basis (and intends to pay the relevant APC), the terms of reuse of such Open Access shall be governed by a Creative Commons licence – details of these licences and which Creative Commons licence will apply to this Work are set out in our licence referred to above.

Other than as permitted in any relevant BMJ Author's Self Archiving Policies, I confirm this Work has not been accepted for publication elsewhere, is not being considered for publication elsewhere and does not duplicate material already published. I confirm all authors consent to publication of this Work and authorise the granting of this licence.

For peer review only

Improving outcomes for primary school children at risk of cerebral visual impairments (The CVI Project): study protocol for the process evaluation of a feasibility cluster-randomised controlled trial

Anna Pease[1], Trudy Goodenough[1], Parisa Sinai[1], Katie Breheny[2], Rose Watanabe[1], Cathy Williams[1]

[1] Centre for Academic Child Health, Bristol Medical School, University of Bristol, 1-5 Whiteladies Road, BS8 1NU, Bristol, UK
Population Health Sciences, Bristol Medical School, University of Bristol, Bristol, UK

Word count: 3094 (limit 4000)

Corresponding Author: C Williams, 1-5 Whiteladies Road, Bristol BS8 1NU
Cathy.Williams@bristol.ac.uk

For peer review only

Abstract

Introduction

Brain related visual impairments, also known as Cerebral Visual Impairment or CVI are related to damage or poor function in the vision related areas of the brain. There is broad agreement that CVI is an appropriate term to describe visual impairments that are not accounted for by disorders of the eye or optic nerve but differences remain as to which impairments can be included in this term. The CVI Project is a programme of work that includes the development of a complex intervention to share knowledge with teachers so that they can make both targeted and universal changes to support children with CVI. A feasibility study for a cluster randomised controlled trial to evaluate this intervention is underway. This paper describes the protocol for an accompanying process evaluation to explore how the intervention is implemented and provide context for the interpretation of the feasibility trial outcomes.

Methods and analysis

A logic model has been developed to guide data collection. Both qualitative and quantitative data will be collected to assess the feasibility and acceptability of the intervention, the study design, and explore how any changes that occur are brought about. Interviews with key primary school staff and parents will investigate responses to the intervention and trial processes. Surveys will collect data on intervention implementation and knowledge of CVI. Photographs of classroom walls will document any changes to visual clutter and document analysis will look for changes to school Special Educational Needs and Disabilities (SEND) policies.

Ethics and dissemination

Ethical approval was granted by the University of Bristol Faculty of Health Sciences Ethics Committee. Findings will contribute to the development of a full-scale cluster RCT to assess the effectiveness of the intervention with adequate statistical power. The results will also support the refinement of the intervention, and its underlying theory.

Keywords: Cerebral Visual Impairment, Complex intervention, feasibility trial, process evaluation, child.

Strengths and limitations of this study

- **Intervention developed with input from professionals and families**
- **Novel intervention to improve awareness of CVI and strategies to help**
- **Process evaluation designed to inform future full-scale trial, if warranted**
- **Study designed using programme logic to inform data collection timelines and methods**
- **Ethical approval re-established following COVD-19 related pause in the study**

For peer review only

INTRODUCTION

Damage or poor function in the brain areas related to vision leads to brain-related visual impairments, also known collectively as cerebral visual impairment (CVI) or sometimes in the plural (CVIs) (1, 2). CVI in children can manifest in many ways; for example, being unable to find things in a cluttered page; bumping into people; being unable to copy from the class whiteboard to their own workbooks or difficulties controlling their eye position effectively to keep focused on a task (3). It has been reported that vision problems may be misinterpreted by teachers as behavioural or other difficulties (4) and so children with CVI may not get the help they need. Concern has been raised that missing a diagnosis of CVI can affect mental health(5, 6), and research from a UK birth cohort suggested that children with CVI may experience academic difficulties(7). Research into strategies to support children with CVI has suggested that providing a less cluttered visual environment may help (8, 9).

In preparation for this study, discussions with two parent advisory groups, and with an advisory group comprising local health and education professionals, have reinforced accounts that simple adaptations of the child's visual world can be very helpful. They emphasize children's improved sense of well-being when they can achieve more and are not chastised for behaviour resulting from their CVI. They have told us that access to help is uneven between and within different areas of the country, and therefore is inequitable, and that few teachers, SENCo (Special Educational Needs Co-ordinator) or Learning Support Assistants (LSAs) know about CVI. In general, only children with reduced visual acuity have access to specialist vision support teachers (QTVIs) and so many children with CVI but good visual acuity, are ineligible for their help. A prevalence study conducted by our group has indicated that there are likely to be at least 3% of children with CVI-related vision problems in primary schools in England and that these are largely but not exclusively within the subset of children already identified as needing extra educational help(10).

Aim of the process evaluation

The aim of the CVI Project process evaluation is to understand both the functioning of the trial processes and the intervention itself. This understanding is crucial to the development and decision to proceed to the full trial.

METHODS AND ANALYSIS

Patient and Public Involvement

We have spoken with two groups of parents whose children have special educational needs and/or CVI. They have been consulted at every stage of the research so far and provided guidance on which measures to use with parents, and which topics to include in qualitative interviews. They advised us on the need to raise awareness in schools about CVI, and we changed our intervention pack to include specific resources to address this. They were clear that teachers should be reimbursed for their time to take part in the study and that we should outline the potential benefits of identifying CVI whereby the right strategies can be put in place, freeing up teacher time not spent trying ineffective interventions. They also advised us that schools might appreciate the benefit of being able to demonstrate to OFSTED (Office for Standards in Education) inspectors that they were implementing strategies to support students who may be struggling with their learning.

For peer review only

The CVI Project Intervention

We have developed an intervention pack which seeks to share knowledge with teachers so that they can make both universal and targeted changes within their school, to better support children with CVI and potentially other children too(9).

The development of the intervention pack is based on recent guidance, including a review of the published literature, using an iterative process of development and refining, involving key stakeholders, developing an underlying theory that takes into account the context and possible real world implementation issues (11). The key elements of the intervention and the hypothesised outcomes are summarised in a Logic model - see Figure 1.

We hypothesize that this multi-faceted approach involving both schools and the hospital service, will improve educational and wellbeing-related outcomes for any child with CVI and potentially for their peers as well. We have therefore devised an information-sharing pack to give schools this information. This includes an overview and explanations of the condition and gives suggestions as to how to make the school more “vision friendly”. Central to this is to use universal approaches wherever possible - for instance decluttering walls and classrooms, using larger font and clear layouts with worksheets. Additional targeted strategies, for use with at-risk children, will also be given, with explanations about who might benefit from these, how they are intended to work and their potential impact. The information included in the intervention pack for teachers is collated from several centres of excellence that have developed materials about CVI, including Bartiméus in the Netherlands (12) and the international TeachCVI initiative (13). The intervention also includes sharing information with the local paediatric ophthalmology clinics about the presentations of and support for children with CVI, so that they are happy to accept referrals of at-risk children. Intervention schools will be able to refer a maximum 5% of children to the paediatric ophthalmology clinic for a functional visual CVI assessment. A printout of the CVI assessment will be available to the family and school with strategies where applicable.

A logic model showing proposed mechanisms of change, impact and outcomes is shown in Figure 1. This model has been refined throughout our intervention development with parents and professionals, and includes consideration of unintended negative consequences of the intervention, or ‘dark logic’, as proposed by Bonell and colleagues (14).

Figure 1: CVI Project Intervention Logic Model

The CVI Project Feasibility Trial

The intervention and trial processes are being tested in a cluster randomised feasibility trial, with nested process and health economic evaluations. The main aim of this trial is to assess the feasibility and acceptability of our intervention and trial methods, to inform the development of a future adequately-powered cluster-randomised controlled trial, if warranted (15). The full protocol for the feasibility trial is presented separately and is summarised here, to give context for the process evaluation, which is described in full below.

The feasibility trial involves mainstream primary schools in three regions of the UK: Gloucester, Hampshire and Somerset. Baseline and follow up data will be collected between September 2019 and February 2021 on students in years 3-5 attending participating schools. In this trial, the primary outcome (child self-reported mental wellbeing) will be assessed in all children who are getting extra help, rather than only in children with confirmed CVI. We will not assess every child in the study for

For peer review only

CVI. This is because in real life this would not be possible and our aim is to evaluate an intervention that could be scaled up for widespread use, if found to be effective. Furthermore, if we examined all children at the study start to identify those who fitted a definition of CVI, or all those with CVI-related vision problems, it would then be unethical not to offer help to identified children and so we would not have a control group. In our earlier study(10), most children with CVI were found within the group getting extra help and these children are easily identifiable by schools (whereas children with confirmed CVI are not). In addition, we also found some children with CVI-related vision problems who were not getting extra help and we hypothesize many children might benefit from some aspects of the intervention e.g. decluttering the classroom to make the visual environment less busy. We will therefore collect data for all children in years 3-5; the primary outcome will be assessed in the children getting extra help, but we will include all children in a secondary outcome analysis.

After baseline data are collected, a statistician separate from the study will randomise the participating schools, with two stratification variables to maintain similar sample sizes in each arm (16) 1) 15% or less of children having extra help or more than 15% of children and 2) size of school (3+ classes per year vs 2 or fewer classes per year, in years 3-5). Schools will be told immediately whether they are allocated to the control group (and will be asked to carry on as normal for the school year) or the intervention group (where they will be invited to implement the CVI intervention pack, described above).

Some of the outcomes of the feasibility study will be assessed using data collected during the study in a process evaluation. The process evaluation will use a mixture of quantitative and qualitative methods for data collection. Complex interventions are described as those which contain several interacting components and may also relate to the implementation of the intervention and its interaction with its context (17). Process evaluations embedded within complex interventions aim to understand the functioning of an intervention by examining implementation, mechanisms of impact and contextual factors (18). There are several frameworks which combine these aspects of process evaluation within feasibility of complex interventions and RCTs; for this study our framework is based on guidance from the Medical Research Council (MRC) (17). We have used this guidance to develop the aims and conceptual framework for the PE. These are described below.

The seven domains to be evaluated as recommended by MRC guidance are as follows:

1. *Implementation of the intervention*

This domain aims to discover what is implemented in practice. We will consider how the CVI Project Intervention Training package is used in each school. We have made suggestions for how schools may wish to implement the intervention (including delivering the presentation at a staff meeting and sharing the resources with class teachers), however we have not been prescriptive about this. Early discussions with teachers in our advisory groups suggested that individual schools would prefer to find their own ways to implement the intervention pack, and that autonomy over this process would be appreciated. We will assess the quality (fidelity) of the intervention and how much of the intervention (dose) was delivered and to whom (reach). We will also determine if there have been any adaptations to the intervention and the reason or purpose of these adaptations. Consideration will also be given to the core components of the intervention, and the barriers and facilitators which influence how individual components of the intervention are selected and delivered.

For peer review only

2. Mechanism of Impact/change

The process evaluation will consider how the intervention as delivered by schools, worked. Research questions will provide information and insights about how the participants, schools and parents responded to and interacted with the intervention. Questions will examine changes in the understanding and knowledge of staff about CVI and how this interacted with their implementation of the intervention, along with exploring the barriers and facilitators they experienced to implementation. Questions will also consider if there were any unintended or unexpected consequences or pathways of the intervention.

3. Programme Differentiation and Usual Practice

The research questions in this domain will examine how the intervention differed from usual practice in the intervention schools. Contamination from intervention to control schools is a potential risk, where schools in the control group make changes related to CVI after hearing about the intervention from a school in the intervention arm. We will seek to assess this with exploratory questions about how and why this occurred and the outcome for control schools.

4. Acceptability of the intervention

This domain will consider the interactions of participants, both school staff and parents with the intervention. The effects of acceptability on implementation of the intervention will be explored. We will consider if acceptability changes across the school year, and how the responses and interactions of staff and parents with the intervention might change the way the intervention is implemented.

5. Sustainability

This domain will consider whether the intervention could become part of normal practice after the feasibility trial was complete. We will assess the fidelity, dose and reach of the intervention in is sustained form. Where it has not been sustained, or there are no plans to continue with the changes after the trial, we will explore why this is. Barriers and facilitators to sustainability will also be explored.

6. Context

Contextual factors may influence the effectiveness of an intervention both directly and indirectly. These are external factors which can impede or strengthen its implementation or effects (MRC). We will consider how the paediatric ophthalmology clinics respond and interact with the intervention, including the referral pathway from schools to HES. In addition, this domain will consider the wider contextual factors which could affect the implementation of the intervention.

7. Trial Process

Understanding the participants responses to and interactions with the trial processes will allow an assessment of how the trial methods might require adjustment or re-design for further work. Understanding the responses of the participants will also provide information about recruitment and continued participation in the trial by both schools, teachers and parents.

Design

The CVI Project is a feasibility cluster randomised study with nested process evaluation and health economic assessment. The process evaluation will use both quantitative and qualitative data sources in order to answer our research questions. We anticipate that all schools will contribute data to the process evaluation, allowing for different experiences with the study between intervention and control arms.

For peer review only

Data sources

We have summarised the research domains, research questions and methods of evaluation in Table 1.

For peer review only

Table 1: Process evaluation research domains and methods of evaluation

**Domain**	**Research questions**<br><br>***Process evaluation framework mapping***	**Informant/source of data**	**Methods**	**Timing of data collection**	**Analysis**
**Implementation of intervention (the information pack)**<br><br>**Intervention Schools n=4**	Which CVI Project information pack components were delivered?<br><br>*Fidelity, dose, reach*	SENCO/head teacher<br>n=4	Brief survey question	Post-intervention	Data recorded and tabulated
	Which resources were used?<br><br>How were these resources used?<br><br>*Fidelity*	SENCO/head teacher<br>n=4	Brief survey questions<br><br>Semi-structured interview	Post-intervention	Data recorded and tabulated<br><br>Thematic analysis
	How was the training delivered? For example: training day, short sessions.<br><br>*Fidelity, adaptation*	SENCO/head teacher<br>n=4	Brief survey questions	Post-intervention	Data recorded and tabulated
	Who received the training?<br><br>*Reach*	SENCO/head teacher<br>n=4	Brief survey questions	Post-intervention	Data recorded and tabulated
	Were adaptations made to resources? How did this affect implementation?<br><br>*Adaptation, Fidelity*	SENCO/head teacher<br>n=4	Semi-structured Interview	Post-intervention	Thematic analysis
	What were the barriers and facilitators to delivering the training?<br><br>*Mediators*	SENCO/head teacher<br>n=4	Semi-structured Interview	Post-intervention	Thematic analysis
**Mechanisms of change of CVI Project intervention**<br><br>**Intervention Schools**	What was the response of staff to the training?<br><br>*Participant responses to and interactions with the CVI Project Intervention*	SENCOs/head teacher classroom teachers, LSA years 3-5<br>n=TBC	Semi-structured Interview	Post-intervention	Thematic Analysis

For peer review only

**n=4**	How did staff knowledge about CVI change in response to training/intervention?<br><br>*Participant responses, reach*	SENCO/head teacher classroom teachers,<br>LSAs years 3-5<br>n=TBC	Bartiméus Centre (Netherlands)<br>Teacher CVI Knowledge Assessment Survey	Brief online survey pre randomisation follow up data collection.	Quantitative analysis
	What were staff and parents' responses to the implementation of the CVI Project intervention?<br><br>*Participant responses to and interactions with the CVI Project Intervention*	SENCO/head teacher classroom teachers, LSA years 3-5<br>n=TBC	Semi-structured Interview	Post- intervention	Thematic analysis
	What changes to school environment took place? E.g. Whole school/class/individual child changes<br><br>*Fidelity, adaptations, and reach*<br>*Context of implementation of intervention*	SENCO/head teacher, classroom teachers, years 3-5<br>n=23 classrooms<br><br>SENCO/head teacher n=4	Photographs taken of study designated viewpoints of each wall in years 3-5 classrooms<br><br>Semi-structured interviews	Pre and post randomisation<br>Pre-follow up data collection<br><br>Post follow up data collection	Quantitative analysis using validated University of Bristol 'clutter' measure<br><br>Thematic analysis
	Were there any unintended/unexpected consequences from the intervention? What were they? How could they be mitigated (if necessary)?<br><br>*Unintended/unexpected pathways or consequences*	SENCO/head teacher, classroom teachers, LSAs years 3-5; parents<br>n=TBC	Semi-structured Interview	Post-intervention	Thematic analysis
	What were the barriers and facilitators for the implementation of the CVI Project intervention?<br><br>*Participant responses to and interactions with the CVI Project Intervention*	SENCO/head teacher, classroom teachers, LSAs years 3-5; parents<br>n=TBC	Semi-structured Interview	Post-intervention	Thematic analysis
**CVI Project intervention differentiation and usual practice**	Was any contamination detected from intervention to control schools?	SENCO/head teacher<br>n=7 (all schools)<br><br>Head teacher/SENCO and classroom teachers, years 3-5	Semi-structured Interview<br><br>Photographs of study designated viewpoints of	Follow up data collection	Thematic analysis

For peer review only

**Intervention schools n=4**<br><br>**Control Schools n=3**	Were there any changes in school SEND documentation over the course of the study that might indicate contamination?<br><br>*Contamination*	n=35 classes (control and intervention schools)<br><br>SEND Policy documents in all schools<br>n=7	each wall in years 3-5 classrooms<br><br>Structured review of schools' SEND documentation	Pre-randomisation and at follow up data collection<br><br>Pre-follow up data collection	Validated University of Bristol 'clutter' measure<br>Quantitative analysis<br><br>Content analysis
	What was different about the CVI Project Intervention when compared to usual practice within the school?<br><br>*Differentiation, mediators to implementation of the intervention*	Head teacher/SENCO and classroom teachers, years 3-5<br>n=TBC	Semi-structured Interview	Follow up data collection	Thematic analysis
**Acceptability of the Intervention**	Was the CVI project intervention acceptable to school staff and parents?<br><br>*Acceptability: participants' responses and interactions with the intervention*	Intervention schools:<br>SENCO/head teacher, classroom teacher/LSA years 3-5, parents<br>n=TBC	Semi-structured Interview	Post Follow-up	Thematic analysis
	What were the experiences of staff and parents of taking part in the CVI project intervention?<br><br>*Participant interactions with the CVI Project Intervention*	Intervention schools:<br>SENCO/head teacher, classroom teachers, LSAs years 3-5; parents<br>n=TBC	Semi-structured Interview	Post Follow-up	Thematic analysis
**Sustainability of the intervention**	Did schools/were schools intending to continue with intervention after the trial period?<br><br>*Sustainability, participants' responses to the intervention, fidelity, quality of sustained use.*	SENCO/head teacher<br><br>n=4	Semi-structured Interview	Post Follow-up	Thematic analysis
	How was the intervention integrated into usual practice?<br><br>Was intervention integrated into SEND policies?	SENCO/head teachers, classroom teachers, years 3-5<br>n=TBC<br><br>SEND policy documents from intervention schools n=4	Semi-structured Interview<br><br>Structured review of SEND policy documents	Post Follow-up<br><br>Pre-randomisation<br>Post Follow-up	Thematic analysis<br><br>Content analysis

For peer review only

	Mechanisms of sustainability of intervention				
	What factors made it more/less likely to be sustained/adopted into usual practice?<br><br>*Barriers and facilitators/mediators to sustainability of the intervention*	SENCO/head teacher<br><br>n=4	Semi-structured Interview	Post Follow-up	Thematic analysis
**Context**<br><br>**Intervention Schools n=4**	What were the experiences of paediatric ophthalmology clinics and orthoptists?<br><br>*Participants responses to the intervention*<br>*Barriers and facilitators to participation in the CVI Project feasibility RCT*	Head orthoptist<br>n=3	Semi-structured Interview	Post Follow-up	Thematic analysis
	How did the referral process from schools to paediatric ophthalmology clinics work?<br><br>*Fidelity, adaptations, reach, barriers and facilitators*<br>*Contamination, unintended consequences*	Paediatric ophthalmology clinics<br>n=3<br>SENCO/head teacher<br>n=4	Semi-structured Interview	Post Follow-up	Thematic analysis
	How many children were given study referral letters for their parents?<br>Who were they referred to?<br><br>What were the characteristics of the children who attended the paediatric ophthalmology clinic with a study referral letter?<br><br>*Fidelity, dose, reach*	SENCO/head teacher<br>n=4<br><br>Paediatric ophthalmology clinics<br>n=3	Records kept at each school<br>Schools collate data<br><br>CVI-vision assessment Conducted by orthoptists	Throughout trial	Count and record<br><br>Recorded on University REDCap database accessed via paediatric ophthalmology clinics.
	How did contextual factors affect intervention implementation? (e.g. funding pressures; reorganisation; academisation, Ofsted, SATS)<br><br>*Implementation and mechanisms of impact*	SENCO/head teacher<br>n=4	Semi-structured Interview	Pre-follow up data collection	Thematic analysis

**Trial Process**<br><br>**Intervention and Control Schools n=7**	What did schools think about the recruitment process?<br><br>*Trial methods, acceptability to participants. Participant responses to trial methods Barriers and facilitators*	Head teachers/SENCOs in control and intervention schools<br><br>n=7	Semi-structured Interview	Post baseline data collection	Thematic analysis
	What was the initial recruitment rate?<br><br>*Recruitment data*	Demographic data from publicly available government schools data base	Extraction of demographic data	Post recruitment	Descriptive data summaries
	What was the dropout rate of schools from the research? What reasons were given?<br><br>*Participant responses and interactions with the trial processes*	Recorded as part of trial routine data collection	Quantitative data with some free text/coded responses for reasons	Ongoing throughout study period	Count and record
	How many parents withdrew their children from the study? What reasons were given?<br><br>*Participant responses and interactions with the trial processes*	Recorded as part of trial routine data collection	Quantitative data with some free text/coded responses for reasons	Ongoing throughout study period	Count and record
	How did schools communicate with parents about trial?<br>*Mechanisms of impact, fidelity, reach*	Examination of school documents/websites/newsletters	Structured review of documents	Ongoing throughout study period	Content analysis
	Acceptability of trial processes: how did schools respond to randomisation? Including the arm of the trial that they were allocated to?<br><br>*Acceptability of trial processes, interactions of participants with trial processes*	SENCO/head teacher in intervention and control schools<br><br>n=7	Semi-structured interview	Post randomisation	Thematic analysis

For peer review only

Surveys

Each intervention school will complete a brief school-level survey at baseline, conducted at the beginning of the qualitative interview. The survey includes 6 questions about the implementation of the intervention pack including how it was delivered, who delivered it, which components of the intervention pack have been used, who received the training, how many referral letters have been sent out and how many students have been identified for referral. Follow up questions will include numbers who have received the intervention, which components have been used, numbers of children identified and referred.

Teacher knowledge of CVI will be assessed before randomisation and at follow up, using the Bartiméus Centre (Netherlands) Teacher CVI Knowledge Assessment Survey (19). All teachers for years 3-5 in all schools will complete these. The survey is comprised of 5 statements about knowledge of CVI (e.g. 'I understand the impact of CVI in daily life') and respondents are asked to rate whether they feel each statement is true for them on a scale of 0-10.

Qualitative interviews with school representative (all schools)

One key representative from each school (usually the SENCO or head teacher) will take part in a semi-structured interview at baseline following randomisation, (after the intervention has been delivered in intervention schools) and at follow up. The initial interview will cover school ethos, experiences with data collection, trial processes (including randomisation) and any changes during the study period. Additional topics for intervention schools will include responses to the intervention pack, resources implemented, feedback from staff, monitoring and recording of changes.

Qualitative interviews with school staff (intervention schools only)

School staff will be invited to take part in a semi-structured interview post intervention implementation, to share their experiences of the CVI Project intervention. The interviews will explore reactions to the intervention materials, how they have been used, views and opinions on their use and usefulness, changes to provision, implementation of both universal and targeted strategies and any feedback on the data collection methods used in the trial. Interviews with staff will take place as the intervention is being used at the school.

Qualitative interviews with parents (all schools)

Parents who returned 'consent to contact' forms will be contacted and invited to take part in a semi-structured interview about their experiences with the study. Interviews will explore their experiences with the data collection, surveys and any thoughts they had about randomisation. Interviews will also cover any changes during the study period and what these can be attributed to, and any referral processes and experiences with the paediatric ophthalmology clinics. Any positive, negative or unintended consequences will be discussed.

Qualitative interviews with paediatric ophthalmology clinic staff

Paediatric ophthalmology clinic staff with involvement in the study will be invited to take part in a semi-structured interview to explore their experiences with referrals from schools. The topics covered in these interviews will include the referral process, characteristics of children who are referred, communication between school, family and hospital unit.

Classroom Clutter (all schools)

For peer review only

We will ask schools to take photographs of each wall in the classrooms used by years 3-5 in each school, pre-randomisation and again at follow up. Photographs of the classrooms at the start and end of the study period will be compared and scored subjectively for the amount of clutter, by observers unaware of the time they were taken. We will also pilot using an adaptation of a visual clutter scoring system originally used in a study of how the visual characteristics of a natural scene affect bird and human search behaviour(20).

School document analysis

We will investigate whether there any changes in school SEND documentation over the course of the study that might indicate contamination by conducting a structured review of all school's SEND policy documentation pre-randomisation and at follow up. They will be studied for any changes made during the study period. Changes found will be included as topics for discussion with SENCo and staff interviews.

Analysis of qualitative data

With consent, the semi-structured interviews will be audio recorded and then transcribed verbatim, anonymised, checked for accuracy and imported into NVivo12(21). Thematic analysis using a continuous comparative method will be used to identify recurrent ideas and patterns within the data (22). Transcripts will be independently coded by two researchers to enhance rigour by cross checking the coding strategy and early development of themes. During analysis, themes will be discussed among the team until a final consensus on the nature and phrasing of key themes and subthemes is reached. Anonymised quotes will be presented to illustrate the main themes.

Analysis of quantitative data

Quantitative survey data will be presented as counts and percentages, as appropriate. String variables will be coded into categories. The CVI knowledge survey will be scored and comparisons made between baseline and follow up. Survey data will be compared between intervention and control schools, but this will be exploratory and will be reported descriptively as the study is not powered to detect an effect size (15).

Photographs of the classroom setting will be analysed using a clutter rating scale, developed at baseline with independent blind ratings by an academic not connected with the study, but with expertise in this area.

School policy documents will be subject to content analysis by the study team, using a data extraction table to identify any differences between schools at the beginning and end of the study period.

ETHICS and HRA

Ethical approval for the study was obtained from the University of Bristol Faculty of Health Sciences Ethics Committee (FREC Ref: 89144). The study is registered on the International Standard Randomised Controlled Trial Number registry (registration number: ISRCTN13762177). Approval from the Health Research Authority (HRA- Ref 19/HRA/6124) has been obtained.

Full details of consent procedures are described in our feasibility trial protocol paper (in preparation), but in brief: Head teachers agree to participate on behalf of the children and staff in his/her school, information sheets will be provided to school staff and parents, parents will be able to withdraw their child's data by completing a non-agreement form. Verbal agreement for child completed measures will also be sought and questionnaires will not be completed by any child who

For peer review only

does not wish to do so. A link to the full Privacy notice online (23) will be provided to all parents and school staff.

Conclusions

We have presented a protocol for a detailed process evaluation of a complex information sharing intervention to improve the mental well-being of primary school children with CVI. Development of an intervention to support children with CVI has been requested by parents (24), and we have developed it with their input. This feasibility study will assess whether and how a future trial should establish effectiveness of the intervention with feedback from this process evaluation. We have addressed key domains as per MRC guidance, and have developed our process evaluation research questions around these aims. Data gathered will help us to interpret the findings from the trial, but importantly, will help to refine the design, content and delivery of the intervention. We have also included data to improve trial processes and communication between schools and the research team in a future full-scale trial. Given that this is a process evaluation for a feasibility trial, we will not be powered to interpret the primary outcome differences between intervention and control, and the data gathered as part of the process evaluation will be limited in explaining any differences in the main feasibility study outcomes.

TRIAL STATUS

Favourable opinion by the University of Bristol faculty ethics committee was obtained on 12th August 2019 (Ref: 89144). We completed recruitment and baseline data collection from participating schools in all areas. We have recruited 7 schools to the feasibility trial and completed baseline trial data collection in February 2020. Four schools have been allocated to the intervention arm and three to control. Due to the COVID-19 global pandemic and school closures, we paused the study from March - September 2020. We have since obtained ethical approval for minor amendments relating to adjustments for a 'COVID-secure' restart to the study on September 1st 2020, with an extension to the study period until April 30th 2021. We will describe all COVID related changes to the study in our final results paper, as these relate to the findings of our feasibility study.

FUNDING AND SPONSORSHIP

University of Bristol provides Sponsorship for this study. The study is funded by the National Institute for Health Research (NIHR) Senior Research Fellowship ref: SRF-2015-08-005. The views expressed in this publication are those of the authors and not necessarily those of the NIHR, NHS or the UK Department of Health and Social Care.

Author contributions

CW led the development of the research including the design of the intervention and trial methods, with support from AP, TG and PS. RW provided data management and systems support. PS provided governance support. AP and TG led on the design of the process evaluation and associated research methodology. KB led the development of the health economics aspect of the process evaluation. All authors contributed to the final manuscript providing comments and text for the final submitted draft.

For peer review only

Acknowledgements

We are grateful to Ms Mai Baquedano for her support with online consent and questionnaires, via the REDCap system. We are also grateful to our family and professionals advisory group members who provided valuable insight into recruitment and data collection strategies, and input into the intervention delivery.

Competing interests

All the authors declare they have no competing interests

REFERENCES

1. Sakki HEA, Dale NJ, Sargent J, Perez-Roche T, Bowman R. Is there consensus in defining childhood cerebral visual impairment? A systematic review of terminology and definitions. The British journal of ophthalmology. 2018;102(4):424-32.
2. Dutton GN. Cognitive vision, its disorders and differential diagnosis in adults and children: knowing where and what things are. Eye (London, England). 2003;17(3):289-304.
3. Dutton GN, Jacobson LK. Cerebral visual impairment in children. Seminars in neonatology : SN. 2001;6(6):477-85.
4. Swift S, Davidson R, Weems L. Cortical Visual Impairment: Presentation Intervention and Prognosis in Educational Settings. TEACHING Exceptional Children PLus. 2008;4(5):1-14.
5. Lueck A, Dutton G. Vision and the Brain: AFB Press; 2015.
6. Murphy M, Fonagy P. Chief Medical Officers Report 2012. Chapter 10. Mental health problems in children and young people. In: Health UgDo, editor.: UK Government; 2012.
7. Williams C, Northstone K, Sabates R, Feinstein L, Emond A, Dutton GN. Visual perceptual difficulties and under-achievement at school in a large community-based sample of children. PLoS One. 2011;6(3):e14772.
8. McDowell N, Budd JJJoVI, Blindness. The perspectives of teachers and paraeducators on the relationship between classroom clutter and learning experiences for students with cerebral visual impairment. 2018;112(3):248-60.
9. Fisher AV, Godwin KE, Seltman H. Visual environment, attention allocation, and learning in young children: when too much of a good thing may be bad. Psychol Sci. 2014;25(7):1362-70.
10. Williams C, Pease A, Warnes P, Harrison S, Pilon F, Hyvarinen J, et al. Cerebral visual impairment (CVI)-related vision problems in children attending primary schools in England: Cross-sectional survey with nested case-finding study. Developmental Medicine & Child Neurology. 2020;in press.
11. O'Cathain A, Croot L, Duncan E, Rousseau N, Sworn K, Turner KM, et al. Guidance on how to develop complex interventions to improve health and healthcare. 2019;9(8):e029954.
12. Landis JR, Koch GG. The measurement of observer agreement for categorical data. Biometrics. 1977;33(1):159-74.
13. B&H The National Institute for the Blind Visually Impaired and Deafblind in Iceland. TeachCVI [Available from: https://www.teachcvi.net/.
14. Bonell C, Jamal F, Melendez-Torres G, Cummins SJJECH. 'Dark logic': theorising the harmful consequences of public health interventions. 2015;69(1):95-8.
15. Eldridge SM, Lancaster GA, Campbell MJ, Thabane L, Hopewell S, Coleman CL, et al. Defining feasibility and pilot studies in preparation for randomised controlled trials: development of a conceptual framework. PloS one. 2016;11(3):e0150205.

16. Treasure T, MacRae KD. Minimisation: the platinum standard for trials?: randomisation doesn't guarantee similarity of groups; minimisation does. British Medical Journal Publishing Group; 1998.
17. Moore G AS, Barker M, Bond L, Bonell C, Hardeman W, Moore L, O'Cathain A, Tinati T, Wight D, Baird J. Process evaluation of complex interventions: Medical Research Council guidance. MRC Population Health Science Research Network, London, 2014 Process evaluation of complex interventions: Medical Research Council guidance. London: MRC Population Health Science Research Network; 2014.
18. Grant A, Treweek S, Dreischulte T, Foy R, Guthrie BJT. Process evaluations for cluster-randomised trials of complex interventions: a proposed framework for design and reporting. 2013;14(1):15.
19. Pilon-Kamsteeg F, Dekker-Pap MJ, de Wit GC, van Genderen MMJBJoVI. CVI Experience toolbox: Simulation of visual processing difficulties. 2019;37(3):248-57.
20. Xiao F, Cuthill IC. Background complexity and the detectability of camouflaged targets by birds and humans. Proceedings of the Royal Society B: Biological Sciences. 2016;283(1838):20161527.
21. Ltd. QIP. NVivo (Version 12) 2018 [Available from: https://www.qsrinternational.com/nvivo-qualitative-data-analysis-software/home.
22. Braun V, Clarke V. Thematic analysis. 2012.
23. The CVI Project Feasibility Study Data Security and Privacy Notice [Available from: http://www.thecviproject.co.uk/privacy/.
24. Jackel B, Wilson M, Hartmann EJJoVI, Blindness. A survey of parents of children with cortical or cerebral visual impairment. 2010;104(10):613-23.

For peer review only

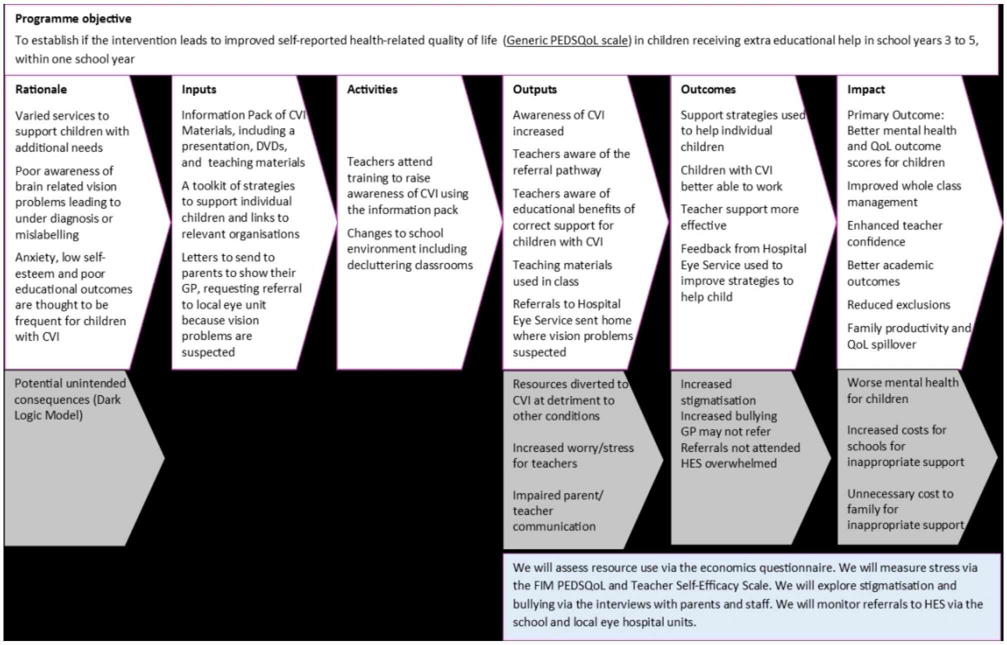
Programme objective
To establish if the intervention leads to improved self-reported health-related quality of life (Generic PEDSQoL scale) in children receiving extra educational help in school years 3 to 5, within one school year
Rationale
Varied services to support children with additional needs
Poor awareness of brain related vision problems leading to under diagnosis or mislabelling
Anxiety, low self-esteem and poor educational outcomes are thought to be frequent for children with CVI
Inputs
Information Pack of CVI Materials, including a presentation, DVDs, and teaching materials
A toolkit of strategies to support individual children and links to relevant organisations
Letters to send to parents to show their GP, requesting referral to local eye unit because vision problems are suspected
Activities
Teachers attend training to raise awareness of CVI using the information pack
Changes to school environment including decluttering classrooms
Outputs
Awareness of CVI increased
Teachers aware of the referral pathway
Teachers aware of educational benefits of correct support for children with CVI
Teaching materials used in class
Referrals to Hospital Eye Service sent home where vision problems suspected
Outcomes
Support strategies used to help individual children
Children with CVI better able to work
Teacher support more effective
Feedback from Hospital Eye Service used to improve strategies to help child
Impact
Primary Outcome: Better mental health and QoL outcome scores for children
Improved whole class management
Enhanced teacher confidence
Better academic outcomes
Reduced exclusions
Family productivity and QoL spillover
Potential unintended consequences (Dark Logic Model)
Resources diverted to CVI at detriment to other conditions
Increased worry/stress for teachers
Impaired parent/ teacher communication
Increased stigmatisation
Increased bullying
GP may not refer
Referrals not attended
HES overwhelmed
Worse mental health for children
Increased costs for schools for inappropriate support
Unnecessary cost to family for inappropriate support
We will assess resource use via the economics questionnaire. We will measure stress via the FIM PEDSQoL and Teacher Self-Efficacy Scale. We will explore stigmatisation and bullying via the interviews with parents and staff. We will monitor referrals to HES via the school and local eye hospital units.

Supplementary materials

A. Teacher CVI knowledge assessment survey

	0	1	2	3	4	5	6	7	8	9	10
1. I know everything about CVI	Not at all										Very much so
2. I understand how complicated the cerebral visual system is	Not at all										Very much so
3. I understand the impact of CVI in daily life	Not at all										Very much so
4. I can explain the impact of CVI in daily life to others.	Not at all										Very much so
5. I know how to adjust the environment of a child with CVI	Not at all										Very much so

For peer review only

B. School staff Interview Topic Guides (Control and Intervention)

CONTROL SCHOOLS TOPIC GUIDE

Introduction

- Check participant is still happy to be involved with the research
- Explain recording device and confidentiality
- Explain purpose of the interview and that participant does not have to talk about anything they feel uncomfortable with and can stop at any time for any reason

Your role

Topics to include:

- Years in role
- Involvement with SEN
- Brief School ethos overall

Experiences with the CVI Project survey collection

Topics/questions to include:

- What was it that first interested you in the study?
- How has communication been with the study team? Anything that we could have done better or made it easier for you (as a member of staff?)
- What did you think about the research agreement process?
- Data collection-how did you feel about that?
- Were the questionnaires? acceptable? Interesting? Too long? Too short?
- Randomisation experiences and feelings
- Classroom photographs

Changes during study period

Topics to include:

- Changes to provision, SEN or other
- How are changes monitored and recorded?
- Are they related to the study or not?
- Changes to policy documents -where did those changes come from? And how does that impact on your practice
- Positive, negative or unintended consequences of trial participation

INTERVENTION SCHOOLS TOPIC GUIDE

Introduction

- Check participant is still happy to be involved with the research
- Explain recording device and confidentiality
- Explain purpose of the interview and that participant does not have to talk about anything they feel uncomfortable with and can stop at any time for any reason

Your role

Topics to include:

- Years in role
- Involvement with SEN
- Brief School ethos overall

Brief Survey Questions

1. **How was the training delivered?**
2. **Who delivered the training?**
3. **Which components of the intervention pack were used?**
4. **Who received the training? How many people in each group and in total?**
5. **How many referral letters sent out to parents if any so far?**
6. **How many students/children identified as would benefit form a referral so far?**

Experiences with the CVI Project survey collection

Topics/questions to include:

- What was it that first interested you in the study?
- How has communication been with the study team? Anything that we could have done better or made it easier for you (as a member of staff?)
- What did you think about the research agreement process?
- Data collection-how did you feel about that?
- Were the questionnaires? acceptable? Interesting? Too long? Too short?
- Randomisation experiences and feelings
- Classroom photographs

Changes during study period

Topics to include:

- **The intervention pack**: initial thoughts about the content and presentation?
- **Training provided**, scope, reach, whose responsibility was it?
- **The intervention**
    - Time and impact on teachers/school/yrs 3, 4 and 5 up to July 2020; yrs 4, 5 and 6 from September 2020
    - What was engagement like from staff?
    - Use in real life, which strategies were used and for who? How did that go?
    - Changes to provision, SEN or other
    - How were changes monitored and recorded?

For peer review only

  - Referrals to eye service experiences, process, feedback, changes, would onsite assessments be better?
  - Changes to policy documents -where did those changes come from? Any intention to change policy in future following intervention?
- Positive, negative or unintended consequences of trial participation
- Accessing specialist provision post-covid, how and source of provision/support

C. Parent interview topic guide (Control and Intervention)

Introduction

- Check participant is still happy to be involved with the research
- Explain recording device and confidentiality
- Explain purpose of the interview and that participant does not have to talk about anything they feel uncomfortable with and can stop at any time for any reason

Your role

Topics to include:

- Describe your children
- Involvement with SEN – existing service use, referrals and stage of process.
- School ethos overall

Experiences with the CVI Project survey collection

Topics to include:

- Data collection: paper forms, returning process, any problems?
- Surveys - acceptable? Interesting? Too long? Too short?
- Randomisation experiences and feelings
- Anything not clear or troubling in surveys?

Interview part 2: Changes during study period

Topics to include:

- Control schools:
  - Changes to provision, SEN or other
  - Did you notice any changes in what is provided for your child at school?
  - Are they related to the study or not? eg classroom changes?
  - Did this impact on anything at home?
  - Positive, negative or unintended consequences of trial participation

- Intervention schools:
  - Changes to provision, SEN or other
  - Did you notice any changes in what is provided for your child at school?
  - Are they related to the study or not? eg classroom changes?
  - Did this impact on anything at home?
  - Positive, negative or unintended consequences of trial participation
  - Referrals to eye service experiences, process, feedback, changes, useful?

For peer review only

D. Hospital eye-service staff interview topic guide

Introduction

- Check participant is still happy to be involved with the research
- Explain recording device and confidentiality
- Explain purpose of the interview and that participant does not have to talk about anything they feel uncomfortable with and can stop at any time for any reason

Your role

Topics to include:

- Years in role
- Involvement with SEN
- Involvement with CVI –previous experience of managing children with CVI

Experiences with the CVI project referral process

Topics to include:

- How did the referral process from schools to eye unit work?
- Timing of referrals, accuracy, ability to book appointments
- Managing additional workload
- Scaling up of intervention if helpful - potential barriers/problems

Experiences with the CVI Project data collection

Topics to include:

- Training to do the visual assessments, experiences and opinions
- Patient expectations for referrals, problems identified, any diagnoses?
- Anything you would change in the research data collection (the Looking Activities)?
- Data entry using REDCap, easy to use?

Feedback to families and schools

Topics to include:

- Parent/carer reactions to appointments and feedback
- Any school responses or contact

Overall experience

Topics to include:

- Positive, negative or unintended consequences of trial participation

For peer review only

BMJ Open

Improving outcomes for primary school children at risk of cerebral visual impairments (The CVI Project): study protocol for the process evaluation of a feasibility cluster-randomised controlled trial

Journal:	*BMJ Open*
Manuscript ID	bmjopen-2020-044856.R2
Article Type:	Protocol
Date Submitted by the Author:	04-Mar-2021
Complete List of Authors:	Pease, Anna; University of Bristol, Bristol Medical School<br>Goodenough, Trudy; University of Bristol, Bristol Medical School<br>Sinai, Parisa; University of Bristol, Bristol Medical School<br>Breheny, Katie; University of Bristol, Bristol Medical School<br>Watanabe, Rose; University of Bristol, Bristol Medical School<br>Williams, Cathy; University of Bristol, Bristol Medical School
**Primary Subject Heading**:	Ophthalmology
Secondary Subject Heading:	Paediatrics, Research methods
Keywords:	EPIDEMIOLOGY, Developmental neurology & neurodisability < PAEDIATRICS, Paediatric ophthalmology < OPHTHALMOLOGY

SCHOLARONE™
Manuscripts

I, the Submitting Author has the right to grant and does grant on behalf of all authors of the Work (as defined in the below author licence), an exclusive licence and/or a non-exclusive licence for contributions from authors who are: i) UK Crown employees; ii) where BMJ has agreed a CC-BY licence shall apply, and/or iii) in accordance with the terms applicable for US Federal Government officers or employees acting as part of their official duties; on a worldwide, perpetual, irrevocable, royalty-free basis to BMJ Publishing Group Ltd ("BMJ") its licensees and where the relevant Journal is co-owned by BMJ to the co-owners of the Journal, to publish the Work in this journal and any other BMJ products and to exploit all rights, as set out in our licence.

The Submitting Author accepts and understands that any supply made under these terms is made by BMJ to the Submitting Author unless you are acting as an employee on behalf of your employer or a postgraduate student of an affiliated institution which is paying any applicable article publishing charge ("APC") for Open Access articles. Where the Submitting Author wishes to make the Work available on an Open Access basis (and intends to pay the relevant APC), the terms of reuse of such Open Access shall be governed by a Creative Commons licence – details of these licences and which Creative Commons licence will apply to this Work are set out in our licence referred to above.

Other than as permitted in any relevant BMJ Author's Self Archiving Policies, I confirm this Work has not been accepted for publication elsewhere, is not being considered for publication elsewhere and does not duplicate material already published. I confirm all authors consent to publication of this Work and authorise the granting of this licence.

For peer review only

Improving outcomes for primary school children at risk of cerebral visual impairments (The CVI Project): study protocol for the process evaluation of a feasibility cluster-randomised controlled trial

Anna Pease[1], Trudy Goodenough[1], Parisa Sinai[1], Katie Breheny[2], Rose Watanabe[1], Cathy Williams[1]

[1] Centre for Academic Child Health, Bristol Medical School, University of Bristol, 1-5 Whiteladies Road, BS8 1NU, Bristol, UK
Population Health Sciences, Bristol Medical School, University of Bristol, Bristol, UK

Word count: 3094 (limit 4000)

Corresponding Author: C Williams, 1-5 Whiteladies Road, Bristol BS8 1NU
Cathy.Williams@bristol.ac.uk

For peer review only

Abstract

Introduction

Brain related visual impairments, also known as Cerebral Visual Impairment or CVI are related to damage or poor function in the vision related areas of the brain. There is broad agreement that CVI is an appropriate term to describe visual impairments that are not accounted for by disorders of the eye or optic nerve but differences remain as to which impairments can be included in this term. The CVI Project is a programme of work that includes the development of a complex intervention to share knowledge with teachers so that they can make both targeted and universal changes to support children with CVI. A feasibility study for a cluster randomised controlled trial to evaluate this intervention is underway. This paper describes the protocol for an accompanying process evaluation to explore how the intervention is implemented and provide context for the interpretation of the feasibility trial outcomes.

Methods and analysis

A logic model has been developed to guide data collection. Both qualitative and quantitative data will be collected to assess the feasibility and acceptability of the intervention, the study design, and explore how any changes that occur are brought about. Interviews with key primary school staff and parents will investigate responses to the intervention and trial processes. Surveys will collect data on intervention implementation and knowledge of CVI. Photographs of classroom walls will document any changes to visual clutter and document analysis will look for changes to school Special Educational Needs and Disabilities (SEND) policies.

Ethics and dissemination

Ethical approval was granted by the University of Bristol Faculty of Health Sciences Ethics Committee. Findings will contribute to the development of a full-scale cluster RCT to assess the effectiveness of the intervention with adequate statistical power. The results will also support the refinement of the intervention, and its underlying theory.

Keywords: Cerebral Visual Impairment, Complex intervention, feasibility trial, process evaluation, child.

Strengths and limitations of this study

- **Intervention developed with input from professionals and families**
- **Novel intervention to improve awareness of CVI and strategies to help**
- **Process evaluation designed to inform future full-scale trial, if warranted**
- **Study designed using programme logic to inform data collection timelines and methods**
- **Ethical approval re-established following COVD-19 related pause in the study**

For peer review only

INTRODUCTION

Damage or poor function in the brain areas related to vision leads to brain-related visual impairments, also known collectively as cerebral visual impairment (CVI) (1, 2). CVI in children can manifest in many ways; for example, being unable to find things in a cluttered page; bumping into people; being unable to copy from the class whiteboard to their own workbooks or difficulties controlling their eye position effectively to keep focused on a task (3). It has been reported that vision problems may be misinterpreted by teachers as behavioural, learning or intellectual difficulties (4) and so children with CVI may not get the help they need. Concern has been raised that missing a diagnosis of CVI can affect mental health(5, 6), and research from a UK birth cohort suggested that children with CVI may experience academic difficulties(7). Research into strategies to support children with CVI has suggested that providing a less cluttered visual environment may help (8, 9).

In preparation for this study, discussions with two parent advisory groups, and with an advisory group comprising local health and education professionals, have reinforced accounts that simple adaptations of the child's visual world can be very helpful. They emphasize children's improved sense of well-being when they can achieve more and are not chastised for behaviour resulting from their CVI. They have told us that access to help is uneven between and within different areas of the country, and therefore is inequitable, and that few teachers, SENCo (Special Educational Needs Co-ordinator) or Learning Support Assistants (LSAs) know about CVI. In general, only children with reduced visual acuity have access to specialist vision support teachers (QTVIs) and so many children with CVI but good visual acuity, are ineligible for their help. A prevalence study conducted by our group has indicated that there are likely to be at least 3% of children with CVI-related vision problems in primary schools in England and that these are largely but not exclusively within the subset of children already identified as needing extra educational help(10).

Aim of the process evaluation

The aim of the CVI Project process evaluation is to understand both the functioning of the trial processes and the intervention itself. This understanding is crucial to the development and decision to proceed to the full trial.

METHODS AND ANALYSIS

Patient and Public Involvement

We have spoken with two groups of parents whose children have special educational needs and/or CVI. They have been consulted at every stage of the research so far and provided guidance on which measures to use with parents, and which topics to include in qualitative interviews. During the study design phase, the parents advised us on the need to raise awareness in schools about CVI, and we changed our draft intervention pack to include specific resources to address this. They were clear that teachers should be reimbursed for their time to take part in the study and that we should outline the potential benefits of identifying CVI whereby the right strategies can be put in place, freeing up teacher time not spent trying ineffective interventions. They also advised us that schools might appreciate the benefit of being able to demonstrate to OFSTED (Office for Standards in Education) inspectors that they were implementing strategies to support students who may be struggling with their learning.

For peer review only

The CVI Project Intervention

We have developed an intervention pack which seeks to share knowledge with teachers so that they can make both universal and targeted changes within their school, to better support children with CVI and potentially other children too(9).

The development of the intervention pack is based on recent guidance, including a review of the published literature, using an iterative process of development and refining, involving key stakeholders, developing an underlying theory that takes into account the context and possible real world implementation issues (11). The key elements of the intervention and the hypothesised outcomes are summarised in a Logic model - see Figure 1.

We hypothesize that this multi-faceted approach involving both schools and the hospital service, will improve educational and wellbeing-related outcomes for any child with CVI and potentially for their peers as well. We have therefore devised an information-sharing pack to give schools this information. This includes an overview and explanations of the condition and gives suggestions as to how to make the school more "vision friendly". Central to this is to use universal approaches wherever possible - for instance decluttering walls and classrooms, using larger font and clear layouts with worksheets. Additional targeted strategies, for use with at-risk children, will also be given, with explanations about who might benefit from these, how they are intended to work and their potential impact. The information included in the intervention pack for teachers is collated from several centres of excellence that have developed materials about CVI, including Bartiméus in the Netherlands(12) and the international TeachCVI initiative.(www.Teachcvi,net) (13)The intervention also includes sharing information with the local paediatric ophthalmology clinics about the presentations of and support for children with CVI, so that they are happy to accept referrals of at-risk children. Intervention schools will be able to refer a maximum 5% of children to the paediatric ophthalmology clinic for a functional visual CVI assessment. A printout of the CVI assessment will be available to the family and school with strategies where applicable.

A logic model showing proposed mechanisms of change, impact and outcomes is shown in Figure 1. This model has been refined throughout our intervention development with parents and professionals, and includes consideration of unintended negative consequences of the intervention, or 'dark logic', as proposed by Bonell and colleagues (14).

Figure 1: CVI Project Intervention Logic Model

The CVI Project Feasibility Trial

The intervention and trial processes are being tested in a cluster randomised feasibility trial, with nested process and health economic evaluations. The main aim of this trial is to assess the feasibility and acceptability of our intervention and trial methods, to inform the development of a future adequately-powered cluster-randomised controlled trial, if warranted (15). The full protocol for the feasibility trial is presented separately and is summarised here, to give context for the process evaluation, which is described in full below.

The feasibility trial involves mainstream primary schools in three regions of the UK: Gloucester, Hampshire and Somerset. Baseline and follow up data will be collected on students in years 3-5 attending participating schools.

For peer review only - http://bmjopen.bmj.com/site/about/guidelines.xhtml

In designing the study, we were aware of 2 challenges; firstly, that in real life it would be impractical to examine all children to look for CVI and secondly that even if that were possible, once identified we would need to help the affected children and would not therefore have a control group. However, in our recent study, I(10), we found that most children with CVI were found within the group getting extra help and these children are easily identifiable by schools (whereas children with confirmed CVI are not). In this feasibility trial therefore, the primary outcome (child self-reported mental wellbeing) will be assessed in all children who are getting extra help, rather than only in children with confirmed CVI. We will not assess every child in the study for CVI. (10)In addition, we also found some children with CVI-related vision problems who were not getting extra help and we hypothesize many children might benefit from some aspects of the intervention e.g. decluttering the classroom to make the visual environment less busy. We will therefore collect data for *all* children in years 3-5; the primary outcome of child-reported quality of life will be assessed in the children getting extra help, but we will include all children in an additional analysis using the same outcome.

After baseline data are collected, a statistician separate from the study will randomise the participating schools, with two stratification variables to maintain similar sample sizes in each arm (16) 1) 15% or less of children having extra help or more than 15% of children and 2) size of school (3+ classes per year vs 2 or fewer classes per year, in years 3-5). Schools will be told immediately whether they are allocated to the control group (and will be asked to carry on as normal for the school year) or the intervention group (where they will be invited to implement the CVI intervention pack, described above).

Some of the outcomes of the feasibility study will be assessed using data collected during the study in a process evaluation. The process evaluation will use a mixture of quantitative and qualitative methods for data collection. Complex interventions are described as those which contain several interacting components and may also relate to the implementation of the intervention and its interaction with its context (17). Process evaluations embedded within complex interventions aim to understand the functioning of an intervention by examining implementation, mechanisms of impact and contextual factors (18). There are several frameworks which combine these aspects of process evaluation within feasibility of complex interventions and RCTs; for this study our framework is based on guidance from the Medical Research Council (MRC) (17). We have used this guidance to develop the aims and conceptual framework for this Process Evaluation (PE)These are described below.

The seven domains to be evaluated as recommended by MRC guidance are as follows:

1. *Implementation of the intervention*

This domain aims to discover what is implemented in practice. We will consider how the CVI Project Intervention Training package is used in each school. We have made suggestions for how schools may wish to implement the intervention (including delivering the presentation at a staff meeting and sharing the resources with class teachers), however we have not been prescriptive about this. Early discussions with teachers in our advisory groups suggested that individual schools would prefer to find their own ways to implement the intervention pack, and that autonomy over this process would be appreciated. We will assess the quality (fidelity) of the intervention and how much of the intervention (dose) was delivered and to whom (reach). We will also determine if there have been any adaptations to the intervention and the reason or purpose of these adaptations. Consideration will also be given to the core components of the intervention, and the barriers and facilitators which influence how individual components of the intervention are selected and delivered.

For peer review only

2. Mechanism of Impact/change

The process evaluation will consider how the intervention as delivered by schools, worked. Research questions will provide information and insights about how the participants, schools and parents responded to and interacted with the intervention. Questions will examine changes in the understanding and knowledge of staff about CVI and how this interacted with their implementation of the intervention, along with exploring the barriers and facilitators they experienced to implementation. Questions will also consider if there were any unintended or unexpected consequences or pathways of the intervention.

3. Programme Differentiation and Usual Practice

The research questions in this domain will examine how the intervention differed from usual practice in the intervention schools. Contamination from intervention to control schools is a potential risk, where schools in the control group make changes related to CVI after hearing about the intervention from a school in the intervention arm. We will seek to assess this with exploratory questions about how and why this occurred and the outcome for control schools.

4. Acceptability of the intervention

This domain will consider the interactions of participants, both school staff and parents with the intervention. The effects of acceptability on implementation of the intervention will be explored. We will consider if acceptability changes across the school year, and how the responses and interactions of staff and parents with the intervention might change the way the intervention is implemented.

5. Sustainability

This domain will consider whether the intervention could become part of normal practice after the feasibility trial was complete. We will assess the fidelity, dose and reach of the intervention in is sustained form. Where it has not been sustained, or there are no plans to continue with the changes after the trial, we will explore why this is. Barriers and facilitators to sustainability will also be explored.

6. Context

Contextual factors may influence the effectiveness of an intervention both directly and indirectly. These are external factors which can impede or strengthen its implementation or effects (MRC). We will consider how the paediatric ophthalmology clinics respond and interact with the intervention, including the referral pathway from schools to HES. In addition, this domain will consider the wider contextual factors which could affect the implementation of the intervention.

7. Trial Process

Understanding the participants responses to and interactions with the trial processes will allow an assessment of how the trial methods might require adjustment or re-design for further work. Understanding the responses of the participants will also provide information about recruitment and continued participation in the trial by both schools, teachers and parents.

Design

The CVI Project is a feasibility cluster randomised study with nested process evaluation and health economic assessment. The process evaluation will use both quantitative and qualitative data sources in order to answer our research questions. We anticipate that all schools will contribute data to the process evaluation, allowing for different experiences with the study between intervention and control arms.

For peer review only

Data sources

We have summarised the research domains, research questions and methods of evaluation in Table 1.

For peer review only

Table 1: Process evaluation research domains and methods of evaluation

Domain	Research questions<br><br>***Process evaluation framework mapping***	Informant/source of data	Methods	Timing of data collection	Analysis
**Implementation of intervention (the information pack)**<br><br>**Intervention Schools n=4**	Which CVI Project information pack components were delivered?<br><br>*Fidelity, dose, reach*	SENCO/head teacher<br>n=4	Brief survey question	Post-intervention	Data recorded and tabulated
	Which resources were used?<br><br>How were these resources used?<br><br>*Fidelity*	SENCO/head teacher<br>n=4	Brief survey questions<br><br>Semi-structured interview	Post-intervention	Data recorded and tabulated<br><br>Thematic analysis
	How was the training delivered? For example: training day, short sessions.<br><br>*Fidelity, adaptation*	SENCO/head teacher<br>n=4	Brief survey questions	Post-intervention	Data recorded and tabulated
	Who received the training?<br><br>*Reach*	SENCO/head teacher<br>n=4	Brief survey questions	Post-intervention	Data recorded and tabulated
	Were adaptations made to resources? How did this affect implementation?<br><br>*Adaptation, Fidelity*	SENCO/head teacher<br>n=4	Semi-structured Interview	Post-intervention	Thematic analysis
	What were the barriers and facilitators to delivering the training?<br><br>*Mediators*	SENCO/head teacher<br>n=4	Semi-structured Interview	Post-intervention	Thematic analysis
**Mechanisms of change of CVI Project intervention**<br><br>**Intervention Schools**	What was the response of staff to the training?<br><br>*Participant responses to and interactions with the CVI Project Intervention*	SENCOs/head teacher classroom teachers, LSA years 3-5<br>n=TBC	Semi-structured Interview	Post-intervention	Thematic Analysis

For peer review only

**n=4**	How did staff knowledge about CVI change in response to training/intervention?<br><br>*Participant responses, reach*	SENCO/head teacher classroom teachers,<br>LSAs years 3-5<br>n=TBC	Bartiméus Centre (Netherlands)<br>Teacher CVI Knowledge Assessment Survey	Brief online survey pre randomisation follow up data collection.	Quantitative analysis
	What were staff and parents' responses to the implementation of the CVI Project intervention?<br><br>*Participant responses to and interactions with the CVI Project Intervention*	SENCO/head teacher classroom teachers, LSA years 3-5<br>n=TBC	Semi-structured Interview	Post- intervention	Thematic analysis
	What changes to school environment took place? E.g. Whole school/class/individual child changes<br><br>*Fidelity, adaptations, and reach*<br>*Context of implementation of intervention*	SENCO/head teacher, classroom teachers, years 3-5<br>n=23 classrooms<br><br>SENCO/head teacher n=4	Photographs taken of study designated viewpoints of each wall in years 3-5 classrooms<br><br>Semi-structured interviews	Pre and post randomisation<br>Pre-follow up data collection<br><br>Post follow up data collection	Quantitative analysis using validated University of Bristol 'clutter' measure<br><br>Thematic analysis
	Were there any unintended/unexpected consequences from the intervention? What were they? How could they be mitigated (if necessary)?<br><br>*Unintended/unexpected pathways or consequences*	SENCO/head teacher, classroom teachers, LSAs years 3-5; parents<br>n=TBC	Semi-structured Interview	Post-intervention	Thematic analysis
	What were the barriers and facilitators for the implementation of the CVI Project intervention?<br><br>*Participant responses to and interactions with the CVI Project Intervention*	SENCO/head teacher, classroom teachers, LSAs years 3-5; parents<br>n=TBC	Semi-structured Interview	Post-intervention	Thematic analysis
**CVI Project intervention differentiation and usual practice**	Was any contamination detected from intervention to control schools?	SENCO/head teacher<br>n=7 (all schools)<br><br>Head teacher/SENCO and classroom teachers, years 3-5	Semi-structured Interview<br><br>Photographs of study designated viewpoints of	Follow up data collection	Thematic analysis

For peer review only

**Intervention schools n=4**<br><br>**Control Schools n=3**	Were there any changes in school SEND documentation over the course of the study that might indicate contamination?<br><br>*Contamination*	n=35 classes (control and intervention schools)<br><br>SEND Policy documents in all schools<br>n=7	each wall in years 3-5 classrooms<br><br>Structured review of schools' SEND documentation	Pre-randomisation and at follow up data collection<br><br>Pre-follow up data collection	Validated University of Bristol 'clutter' measure<br>Quantitative analysis<br><br>Content analysis
	What was different about the CVI Project Intervention when compared to usual practice within the school?<br><br>*Differentiation, mediators to implementation of the intervention*	Head teacher/SENCO and classroom teachers, years 3-5<br>n=TBC	Semi-structured Interview	Follow up data collection	Thematic analysis
**Acceptability of the Intervention**	Was the CVI project intervention acceptable to school staff and parents?<br><br>*Acceptability: participants' responses and interactions with the intervention*	Intervention schools: SENCO/head teacher, classroom teacher/LSA years 3-5, parents<br>n=TBC	Semi-structured Interview	Post Follow-up	Thematic analysis
	What were the experiences of staff and parents of taking part in the CVI project intervention?<br><br>*Participant interactions with the CVI Project Intervention*	Intervention schools: SENCO/head teacher, classroom teachers, LSAs years 3-5; parents<br>n=TBC	Semi-structured Interview	Post Follow-up	Thematic analysis
**Sustainability of the intervention**	Did schools/were schools intending to continue with intervention after the trial period?<br><br>*Sustainability, participants' responses to the intervention, fidelity, quality of sustained use.*	SENCO/head teacher<br><br>n=4	Semi-structured Interview	Post Follow-up	Thematic analysis
	How was the intervention integrated into usual practice?<br><br>Was intervention integrated into SEND policies?	SENCO/head teachers, classroom teachers, years 3-5<br>n=TBC<br><br>SEND policy documents from intervention schools n=4	Semi-structured Interview<br><br>Structured review of SEND policy documents	Post Follow-up<br><br>Pre-randomisation<br>Post Follow-up	Thematic analysis<br><br>Content analysis

For peer review only

	Mechanisms of sustainability of intervention				
	What factors made it more/less likely to be sustained/adopted into usual practice?<br><br>*Barriers and facilitators/mediators to sustainability of the intervention*	SENCO/head teacher<br><br>n=4	Semi-structured Interview	Post Follow-up	Thematic analysis
**Context**<br><br>**Intervention Schools n=4**	What were the experiences of paediatric ophthalmology clinics and orthoptists?<br><br>*Participants responses to the intervention*<br>*Barriers and facilitators to participation in the CVI Project feasibility RCT*	Head orthoptist<br>n=3	Semi-structured Interview	Post Follow-up	Thematic analysis
	How did the referral process from schools to paediatric ophthalmology clinics work?<br><br>*Fidelity, adaptations, reach, barriers and facilitators*<br>*Contamination, unintended consequences*	Paediatric ophthalmology clinics<br>n=3<br>SENCO/head teacher<br>n=4	Semi-structured Interview	Post Follow-up	Thematic analysis
	How many children were given study referral letters for their parents?<br>Who were they referred to?<br><br>What were the characteristics of the children who attended the paediatric ophthalmology clinic with a study referral letter?<br><br>*Fidelity, dose, reach*	SENCO/head teacher<br>n=4<br><br>Paediatric ophthalmology clinics<br>n=3	Records kept at each school<br>Schools collate data<br><br>CVI-vision assessment Conducted by orthoptists	Throughout trial	Count and record<br><br>Recorded on University REDCap database accessed via paediatric ophthalmology clinics.
	How did contextual factors affect intervention implementation? (e.g. funding pressures; reorganisation; academisation, Ofsted, SATS)<br><br>*Implementation and mechanisms of impact*	SENCO/head teacher<br>n=4	Semi-structured Interview	Pre-follow up data collection	Thematic analysis

For peer review only

**Trial Process**<br><br>**Intervention and Control Schools n=7**	What did schools think about the recruitment process?<br><br>*Trial methods, acceptability to participants. Participant responses to trial methods Barriers and facilitators*	Head teachers/SENCOs in control and intervention schools<br><br>n=7	Semi-structured Interview	Post baseline data collection	Thematic analysis
	What was the initial recruitment rate?<br><br>*Recruitment data*	Demographic data from publicly available government schools data base	Extraction of demographic data	Post recruitment	Descriptive data summaries
	What was the dropout rate of schools from the research? What reasons were given?<br><br>*Participant responses and interactions with the trial processes*	Recorded as part of trial routine data collection	Quantitative data with some free text/coded responses for reasons	Ongoing throughout study period	Count and record
	How many parents withdrew their children from the study? What reasons were given?<br><br>*Participant responses and interactions with the trial processes*	Recorded as part of trial routine data collection	Quantitative data with some free text/coded responses for reasons	Ongoing throughout study period	Count and record
	How did schools communicate with parents about trial?<br>*Mechanisms of impact, fidelity, reach*	Examination of school documents/websites/newsletters	Structured review of documents	Ongoing throughout study period	Content analysis
	Acceptability of trial processes: how did schools respond to randomisation? Including the arm of the trial that they were allocated to?<br><br>*Acceptability of trial processes, interactions of participants with trial processes*	SENCO/head teacher in intervention and control schools<br><br>n=7	Semi-structured interview	Post randomisation	Thematic analysis

For peer review only

Surveys

Each intervention school will complete a brief school-level survey during the intervention period, conducted at the beginning of the qualitative interview. The survey includes 6 questions about the implementation of the intervention pack including how it was delivered, who delivered it, which components of the intervention pack have been used, who received the training, how many referral letters have been sent out and how many students have been identified for referral. Follow up questions will include numbers who have received the intervention, which components have been used, numbers of children identified and referred.

Teacher knowledge of CVI will be assessed before randomisation and at follow up, using the Bartiméus Centre (Netherlands) Teacher CVI Knowledge Assessment Survey (12). All teachers for years 3-5 in all schools will complete these. The survey is comprised of 5 statements about knowledge of CVI (e.g. 'I understand the impact of CVI in daily life') and respondents are asked to rate whether they feel each statement is true for them on a scale of 0-10.

Qualitative interviews with school representative (all schools)

One key representative from each school (usually the SENCO or head teacher) will take part in a semi-structured interview at baseline following randomisation, (after the intervention has been delivered in intervention schools) and at follow up. The initial interview will cover school ethos, experiences with data collection, trial processes (including randomisation) and any changes during the study period. Additional topics for intervention schools will include responses to the intervention pack, resources implemented, feedback from staff, monitoring and recording of changes.

Qualitative interviews with school staff (intervention schools only)

School staff will be invited to take part in a semi-structured interview post intervention implementation, to share their experiences of the CVI Project intervention. The interviews will explore reactions to the intervention materials, how they have been used, views and opinions on their use and usefulness, changes to provision, implementation of both universal and targeted strategies and any feedback on the data collection methods used in the trial. Interviews with staff will take place as the intervention is being used at the school.

Qualitative interviews with parents (all schools)

Parents who returned 'consent to contact' forms will be contacted and invited to take part in a semi-structured interview about their experiences with the study. Interviews will explore their experiences with the data collection, surveys and any thoughts they had about randomisation. Interviews will also cover any changes during the study period and what these can be attributed to, and any referral processes and experiences with the paediatric ophthalmology clinics. Any positive, negative or unintended consequences will be discussed.

Qualitative interviews with paediatric ophthalmology clinic staff

Paediatric ophthalmology clinic staff with involvement in the study will be invited to take part in a semi-structured interview to explore their experiences with referrals from schools. The topics covered in these interviews will include the referral process, characteristics of children who are referred, communication between school, family and hospital unit.

Classroom Clutter (all schools)

For peer review only

We will ask schools to take photographs of each wall in the classrooms used by years 3-5 in each school, pre-randomisation and again at follow up. Photographs of the classrooms at the start and end of the study period will be compared and scored subjectively for the amount of clutter, by observers unaware of the school or time they were taken. We will also pilot using an adaptation of a visual clutter quantification system originally used in a study of how the visual characteristics of a natural scene affect bird and human search behaviour(19). We anticipate that we will present the results of analyses using the photographs briefly in our results paper and separately in full.

School document analysis

We will investigate whether there any changes in school SEND documentation over the course of the study that might indicate contamination by conducting a structured review of all school's SEND policy documentation pre-randomisation and at follow up. They will be studied for any changes made during the study period. Changes found will be included as topics for discussion with SENCo and staff interviews.

Analysis of qualitative data

With consent, the semi-structured interviews will be audio recorded and then transcribed verbatim, anonymised, checked for accuracy and imported into NVivo12(20). Thematic analysis using a continuous comparative method will be used to identify recurrent ideas and patterns within the data (21). Transcripts will be independently coded by two researchers to enhance rigour by cross checking the coding strategy and early development of themes. During analysis, themes will be discussed among the team until a final consensus on the nature and phrasing of key themes and subthemes is reached. Anonymised quotes will be presented to illustrate the main themes.

Analysis of quantitative data

Quantitative survey data will be presented as counts and percentages, as appropriate. String variables will be coded into categories. The CVI knowledge survey will be scored and comparisons made between baseline and follow up. Survey data will be compared between intervention and control schools, but this will be exploratory and will be reported descriptively as the study is not powered to detect an effect size (15).

Photographs of the classroom setting will be analysed using a clutter rating scale, developed at baseline with independent blind ratings by an academic not connected with the study, but with expertise in this area.

School policy documents will be subject to content analysis by the study team, using a data extraction table to identify any differences between schools at the beginning and end of the study period.

ETHICS and HRA

Ethical approval for the study was obtained from the University of Bristol Faculty of Health Sciences Ethics Committee (FREC Ref: 89144). The study is registered on the International Standard Randomised Controlled Trial Number registry (registration number: ISRCTN13762177). Approval from the Health Research Authority (HRA- Ref 19/HRA/6124) has been obtained.

Full details of consent procedures are described in our feasibility trial protocol paper (in preparation), but in brief: Head teachers agree to participate on behalf of the children and staff in his/her school, information sheets will be provided to school staff and parents, parents will be able to withdraw their child's data by completing a non-agreement form. Verbal agreement for child

For peer review only

completed measures will also be sought and questionnaires will not be completed by any child who does not wish to do so. A link to the full Privacy notice online (22)will be provided to all parents and school staff.

Conclusions

We have presented a protocol for a detailed process evaluation of a complex information sharing intervention to improve the mental well-being of primary school children with CVI. Development of an intervention to support children with CVI has been requested by parents (23), and we have developed it with their input. This feasibility study will assess whether and how a future trial should establish effectiveness of the intervention with feedback from this process evaluation. We have addressed key domains as per MRC guidance, and have developed our process evaluation research questions around these aims. Data gathered will help us to interpret the findings from the trial, but importantly, will help to refine the design, content and delivery of the intervention. We have also included data to improve trial processes and communication between schools and the research team in a future full-scale trial. Given that this is a process evaluation for a feasibility trial, we will not be powered to interpret the primary outcome differences between intervention and control, and the data gathered as part of the process evaluation will be limited in explaining any differences in the main feasibility study outcomes.

TRIAL STATUS

Favourable opinion by the University of Bristol faculty ethics committee was obtained on 12th August 2019 (Ref: 89144). We completed recruitment and baseline data collection from participating schools in all areas. We have recruited 7 schools to the feasibility trial and completed baseline trial data collection in February 2020. Four schools have been allocated to the intervention arm and three to control. Due to the COVID-19 global pandemic and school closures, we paused the study from March - September 2020. We have since obtained ethical approval for minor amendments relating to adjustments for a 'COVID-secure' restart to the study on September 1st 2020, with an extension to the study period until April 30th 2021. We will describe all COVID related changes to the study in our final results paper, as these relate to the findings of our feasibility study.

FUNDING AND SPONSORSHIP

University of Bristol provides Sponsorship for this study. The study is funded by the National Institute for Health Research (NIHR) Senior Research Fellowship ref: SRF-2015-08-005. The views expressed in this publication are those of the authors and not necessarily those of the NIHR, NHS or the UK Department of Health and Social Care.

Author contributions

CW led the development of the research including the design of the intervention and trial methods, with support from AP, TG and PS. RW provided data management and systems support. PS provided governance support. AP and TG led on the design of the process evaluation and associated research methodology. KB led the development of the health economics aspect of the process evaluation. All authors contributed to the final manuscript providing comments and text for the final submitted draft.

For peer review only

Acknowledgements

We are grateful to Ms Mai Baquedano for her support with online consent and questionnaires, via the REDCap system. We are also grateful to our family and professionals advisory group members who provided valuable insight into recruitment and data collection strategies, and input into the intervention delivery.

Competing interests

All the authors declare they have no competing interests

REFERENCES

1. Sakki HEA, Dale NJ, Sargent J, Perez-Roche T, Bowman R. Is there consensus in defining childhood cerebral visual impairment? A systematic review of terminology and definitions. The British journal of ophthalmology. 2018;102(4):424-32.
2. Dutton GN. Cognitive vision, its disorders and differential diagnosis in adults and children: knowing where and what things are. Eye (Lond). 2003;17(3):289-304.
3. Dutton GN, Jacobson LK. Cerebral visual impairment in children. Seminars in neonatology : SN. 2001;6(6):477-85.
4. Swift S, Davidson R, Weems L. Cortical Visual Impairment: Presentation Intervention and Prognosis in Educational Settings. TEACHING Exceptional Children PLus. 2008;4(5):1-14.
5. Lueck A, Dutton G. Vision and the Brain: AFB Press; 2015.
6. Murphy M, Fonagy P. Chief Medical Officers Report 2012. Chapter 10. Mental health problems in children and young people. In: Health UgDo, editor.: UK Government; 2012.
7. Williams C, Northstone K, Sabates R, Feinstein L, Emond A, Dutton GN. Visual perceptual difficulties and under-achievement at school in a large community-based sample of children. PLoS One. 2011;6(3):e14772.
8. McDowell N, Budd B J VI and Blindness. The perspectives of teachers and paraeducators on the relationship between classroom clutter and learning experiences for students with cerebral visual impairment. 2018;112(3):248-60.
9. Fisher AV, Godwin KE, Seltman H. Visual environment, attention allocation, and learning in young children: when too much of a good thing may be bad. Psychol Sci. 2014;25(7):1362-70.
10. Williams C, Pease A, Warnes P, Harrison S, Pilon F, Hyvarinen J, et al. Cerebral visual impairment (CVI)-related vision problems in children attending primary schools in England: Cross-sectional survey with nested case-finding study. Developmental Medicine & Child Neurology. 2020;in press.
11. O'Cathain A, Croot L, Duncan E, Rousseau N, Sworn K, Turner KM, et al. Guidance on how to develop complex interventions to improve health and healthcare. 2019;9(8):e029954.
12. Pilon-Kamsteeg F, Dekker-Pap MJ, de Wit GC, van Genderen MMJBJoVI. CVI Experience toolbox: Simulation of visual processing difficulties. 2019;37(3):248-57.
13. B&H The National Institute for the Blind Visually Impaired and Deafblind in Iceland. TeachCVI [Available from: https://www.teachcvi.net/.
14. Bonell C, Jamal F, Melendez-Torres G, Cummins SJJECH. 'Dark logic': theorising the harmful consequences of public health interventions. 2015;69(1):95-8.

15. Eldridge SM, Lancaster GA, Campbell MJ, Thabane L, Hopewell S, Coleman CL, et al. Defining feasibility and pilot studies in preparation for randomised controlled trials: development of a conceptual framework. PloS one. 2016;11(3):e0150205.
16. Treasure T, MacRae KD. Minimisation: the platinum standard for trials?: randomisation doesn't guarantee similarity of groups; minimisation does. British Medical Journal Publishing Group; 1998.
17. Moore G AS, Barker M, Bond L, Bonell C, Hardeman W, Moore L, O'Cathain A, Tinati T, Wight D, Baird J. Process evaluation of complex interventions: Medical Research Council guidance. MRC Population Health Science Research Network, London, 2014 Process evaluation of complex interventions: Medical Research Council guidance. London: MRC Population Health Science Research Network; 2014.
18. Grant A, Treweek S, Dreischulte T, Foy R, Guthrie BJT. Process evaluations for cluster-randomised trials of complex interventions: a proposed framework for design and reporting. 2013;14(1):15.
19. Xiao F, Cuthill IC. Background complexity and the detectability of camouflaged targets by birds and humans. Proceedings of the Royal Society B: Biological Sciences. 2016;283(1838):20161527.
20. Ltd. QIP. NVivo (Version 12) 2018 [Available from: https://www.qsrinternational.com/nvivo-qualitative-data-analysis-software/home.
21. Braun V, Clarke V. Thematic analysis. 2012.
22. The CVI Project Feasibility Study Data Security and Privacy Notice [Available from: http://www.thecviproject.co.uk/privacy/.
23. Jackel B, Wilson M, Hartmann EJJoVI, Blindness. A survey of parents of children with cortical or cerebral visual impairment. 2010;104(10):613-23.

For peer review only

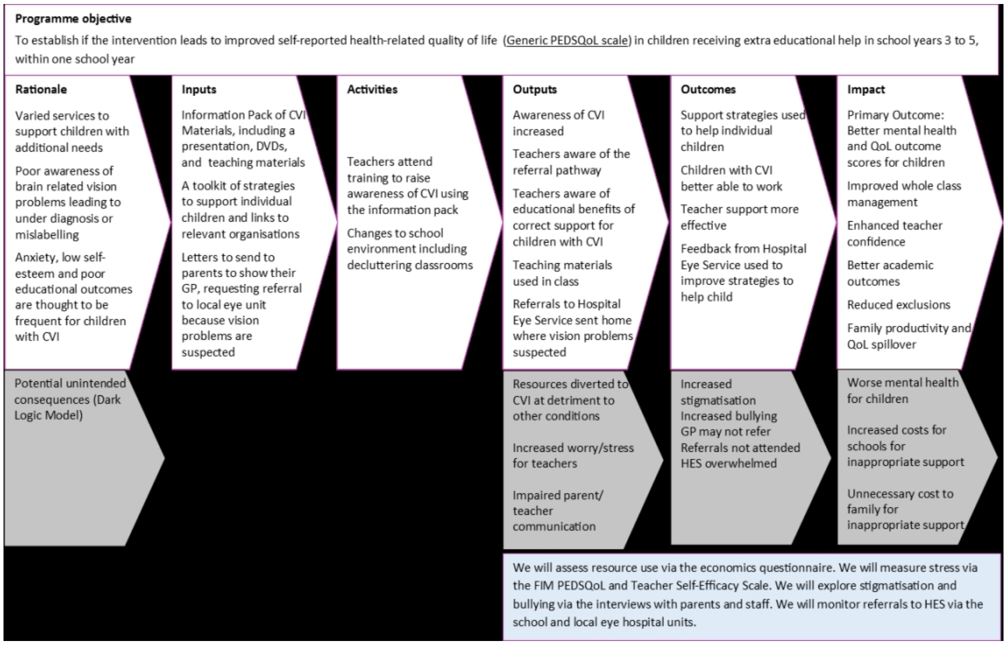
Programme objective
To establish if the intervention leads to improved self-reported health-related quality of life (Generic PEDSQoL scale) in children receiving extra educational help in school years 3 to 5, within one school year
Rationale
Varied services to support children with additional needs
Poor awareness of brain related vision problems leading to under diagnosis or mislabelling
Anxiety, low self-esteem and poor educational outcomes are thought to be frequent for children with CVI
Inputs
Information Pack of CVI Materials, including a presentation, DVDs, and teaching materials
A toolkit of strategies to support individual children and links to relevant organisations
Letters to send to parents to show their GP, requesting referral to local eye unit because vision problems are suspected
Activities
Teachers attend training to raise awareness of CVI using the information pack
Changes to school environment including decluttering classrooms
Outputs
Awareness of CVI increased
Teachers aware of the referral pathway
Teachers aware of educational benefits of correct support for children with CVI
Teaching materials used in class
Referrals to Hospital Eye Service sent home where vision problems suspected
Outcomes
Support strategies used to help individual children
Children with CVI better able to work
Teacher support more effective
Feedback from Hospital Eye Service used to improve strategies to help child
Impact
Primary Outcome: Better mental health and QoL outcome scores for children
Improved whole class management
Enhanced teacher confidence
Better academic outcomes
Reduced exclusions
Family productivity and QoL spillover
Potential unintended consequences (Dark Logic Model)
Resources diverted to CVI at detriment to other conditions
Increased worry/stress for teachers
Impaired parent/ teacher communication
Increased stigmatisation
Increased bullying
GP may not refer
Referrals not attended
HES overwhelmed
Worse mental health for children
Increased costs for schools for inappropriate support
Unnecessary cost to family for inappropriate support
We will assess resource use via the economics questionnaire. We will measure stress via the FIM PEDSQoL and Teacher Self-Efficacy Scale. We will explore stigmatisation and bullying via the interviews with parents and staff. We will monitor referrals to HES via the school and local eye hospital units.

Supplementary materials

A. Teacher CVI knowledge assessment survey

	0	1	2	3	4	5	6	7	8	9	10
1. I know everything about CVI	Not at all										Very much so
2. I understand how complicated the cerebral visual system is	Not at all										Very much so
3. I understand the impact of CVI in daily life	Not at all										Very much so
4. I can explain the impact of CVI in daily life to others.	Not at all										Very much so
5. I know how to adjust the environment of a child with CVI	Not at all										Very much so

B. School staff Interview Topic Guides (Control and Intervention)

CONTROL SCHOOLS TOPIC GUIDE

Introduction

- Check participant is still happy to be involved with the research
- Explain recording device and confidentiality
- Explain purpose of the interview and that participant does not have to talk about anything they feel uncomfortable with and can stop at any time for any reason

Your role

Topics to include:

- Years in role
- Involvement with SEN
- Brief School ethos overall

Experiences with the CVI Project survey collection

Topics/questions to include:

- What was it that first interested you in the study?
- How has communication been with the study team? Anything that we could have done better or made it easier for you (as a member of staff?)
- What did you think about the research agreement process?
- Data collection-how did you feel about that?
- Were the questionnaires? acceptable? Interesting? Too long? Too short?
- Randomisation experiences and feelings
- Classroom photographs

Changes during study period

Topics to include:

For peer review only

- Changes to provision, SEN or other
- How are changes monitored and recorded?
- Are they related to the study or not?
- Changes to policy documents -where did those changes come from? And how does that impact on your practice
- Positive, negative or unintended consequences of trial participation

INTERVENTION SCHOOLS TOPIC GUIDE

Introduction

- Check participant is still happy to be involved with the research
- Explain recording device and confidentiality
- Explain purpose of the interview and that participant does not have to talk about anything they feel uncomfortable with and can stop at any time for any reason

Your role

Topics to include:

- Years in role
- Involvement with SEN
- Brief School ethos overall

Brief Survey Questions

1. **How was the training delivered?**
2. **Who delivered the training?**
3. **Which components of the intervention pack were used?**
4. **Who received the training? How many people in each group and in total?**
5. **How many referral letters sent out to parents if any so far?**
6. **How many students/children identified as would benefit form a referral so far?**

Experiences with the CVI Project survey collection

Topics/questions to include:

- What was it that first interested you in the study?
- How has communication been with the study team? Anything that we could have done better or made it easier for you (as a member of staff?)
- What did you think about the research agreement process?
- Data collection-how did you feel about that?
- Were the questionnaires? acceptable? Interesting? Too long? Too short?
- Randomisation experiences and feelings
- Classroom photographs

Changes during study period

Topics to include:

- **The intervention pack**: initial thoughts about the content and presentation?
- **Training provided**, scope, reach, whose responsibility was it?
- **The intervention**
    - Time and impact on teachers/school/yrs 3, 4 and 5 up to July 2020; yrs 4, 5 and 6 from September 2020
    - What was engagement like from staff?
    - Use in real life, which strategies were used and for who? How did that go?
    - Changes to provision, SEN or other
    - How were changes monitored and recorded?

For peer review only

    - Referrals to eye service experiences, process, feedback, changes, would onsite assessments be better?
    - Changes to policy documents -where did those changes come from? Any intention to change policy in future following intervention?
- Positive, negative or unintended consequences of trial participation
- Accessing specialist provision post-covid, how and source of provision/support

C. Parent interview topic guide (Control and Intervention)

Introduction

- Check participant is still happy to be involved with the research
- Explain recording device and confidentiality
- Explain purpose of the interview and that participant does not have to talk about anything they feel uncomfortable with and can stop at any time for any reason

Your role

Topics to include:

- Describe your children
- Involvement with SEN – existing service use, referrals and stage of process.
- School ethos overall

Experiences with the CVI Project survey collection

Topics to include:

- Data collection: paper forms, returning process, any problems?
- Surveys - acceptable? Interesting? Too long? Too short?
- Randomisation experiences and feelings
- Anything not clear or troubling in surveys?

Interview part 2: Changes during study period

Topics to include:

- Control schools:
    - Changes to provision, SEN or other
    - Did you notice any changes in what is provided for your child at school?
    - Are they related to the study or not? eg classroom changes?
    - Did this impact on anything at home?
    - Positive, negative or unintended consequences of trial participation

- Intervention schools:
    - Changes to provision, SEN or other
    - Did you notice any changes in what is provided for your child at school?
    - Are they related to the study or not? eg classroom changes?
    - Did this impact on anything at home?
    - Positive, negative or unintended consequences of trial participation
    - Referrals to eye service experiences, process, feedback, changes, useful?

For peer review only

D. Hospital eye-service staff interview topic guide

Introduction

- Check participant is still happy to be involved with the research
- Explain recording device and confidentiality
- Explain purpose of the interview and that participant does not have to talk about anything they feel uncomfortable with and can stop at any time for any reason

Your role

Topics to include:

- Years in role
- Involvement with SEN
- Involvement with CVI –previous experience of managing children with CVI

Experiences with the CVI project referral process

Topics to include:

- How did the referral process from schools to eye unit work?
- Timing of referrals, accuracy, ability to book appointments
- Managing additional workload
- Scaling up of intervention if helpful - potential barriers/problems

Experiences with the CVI Project data collection

Topics to include:

- Training to do the visual assessments, experiences and opinions
- Patient expectations for referrals, problems identified, any diagnoses?
- Anything you would change in the research data collection (the Looking Activities)?
- Data entry using REDCap, easy to use?

Feedback to families and schools

Topics to include:

- Parent/carer reactions to appointments and feedback
- Any school responses or contact

Overall experience

Topics to include:

- Positive, negative or unintended consequences of trial participation

For peer review only

BMJ Open

Improving outcomes for primary school children at risk of cerebral visual impairments (The CVI Project): study protocol for the process evaluation of a feasibility cluster-randomised controlled trial

Journal:	*BMJ Open*
Manuscript ID	bmjopen-2020-044856.R3
Article Type:	Protocol
Date Submitted by the Author:	18-Mar-2021

Complete List of Authors:	Pease, Anna; University of Bristol, Bristol Medical School<br>Goodenough, Trudy; University of Bristol, Bristol Medical School<br>Sinai, Parisa; University of Bristol, Bristol Medical School<br>Breheny, Katie; University of Bristol, Bristol Medical School<br>Watanabe, Rose; University of Bristol, Bristol Medical School<br>Williams, Cathy; University of Bristol, Bristol Medical School

**Primary Subject Heading**:	Ophthalmology
Secondary Subject Heading:	Paediatrics, Research methods
Keywords:	EPIDEMIOLOGY, Developmental neurology & neurodisability < PAEDIATRICS, Paediatric ophthalmology < OPHTHALMOLOGY

SCHOLARONE™
Manuscripts

I, the Submitting Author has the right to grant and does grant on behalf of all authors of the Work (as defined in the below author licence), an exclusive licence and/or a non-exclusive licence for contributions from authors who are: i) UK Crown employees; ii) where BMJ has agreed a CC-BY licence shall apply, and/or iii) in accordance with the terms applicable for US Federal Government officers or employees acting as part of their official duties; on a worldwide, perpetual, irrevocable, royalty-free basis to BMJ Publishing Group Ltd ("BMJ") its licensees and where the relevant Journal is co-owned by BMJ to the co-owners of the Journal, to publish the Work in this journal and any other BMJ products and to exploit all rights, as set out in our licence.

The Submitting Author accepts and understands that any supply made under these terms is made by BMJ to the Submitting Author unless you are acting as an employee on behalf of your employer or a postgraduate student of an affiliated institution which is paying any applicable article publishing charge ("APC") for Open Access articles. Where the Submitting Author wishes to make the Work available on an Open Access basis (and intends to pay the relevant APC), the terms of reuse of such Open Access shall be governed by a Creative Commons licence – details of these licences and which Creative Commons licence will apply to this Work are set out in our licence referred to above.

Other than as permitted in any relevant BMJ Author's Self Archiving Policies, I confirm this Work has not been accepted for publication elsewhere, is not being considered for publication elsewhere and does not duplicate material already published. I confirm all authors consent to publication of this Work and authorise the granting of this licence.

For peer review only

Improving outcomes for primary school children at risk of cerebral visual impairments (The CVI Project): study protocol for the process evaluation of a feasibility cluster-randomised controlled trial

Anna Pease[1], Trudy Goodenough[1], Parisa Sinai[1], Katie Breheny[2], Rose Watanabe[1], Cathy Williams[1]

[1] Centre for Academic Child Health, Bristol Medical School, University of Bristol, 1-5 Whiteladies Road, BS8 1NU, Bristol, UK
[2] Population Health Sciences, Bristol Medical School, University of Bristol, Bristol, UK

Word count: 3672 (limit 4000)

Corresponding Author: C Williams, 1-5 Whiteladies Road, Bristol BS8 1NU
Cathy.Williams@bristol.ac.uk

For peer review only

Abstract

Introduction

Brain related visual impairments, also known as Cerebral Visual Impairment or CVI are related to damage or poor function in the vision related areas of the brain. There is broad agreement that CVI is an appropriate term to describe visual impairments that are not accounted for by disorders of the eye or optic nerve but differences remain as to which impairments can be included in this term. The CVI Project is a programme of work that includes the development of a complex intervention to share knowledge with teachers so that they can make both targeted and universal changes to support children with CVI. A feasibility study for a cluster randomised controlled trial to evaluate this intervention is underway. This paper describes the protocol for an accompanying process evaluation to explore how the intervention is implemented and provide context for the interpretation of the feasibility trial outcomes.

Methods and analysis

A logic model has been developed to guide data collection. Both qualitative and quantitative data will be collected to assess the feasibility and acceptability of the intervention, the study design, and explore how any changes that occur are brought about. Interviews with key primary school staff and parents will investigate responses to the intervention and trial processes. Surveys will collect data on intervention implementation and knowledge of CVI. Photographs of classroom walls will document any changes to visual clutter and document analysis will look for changes to school Special Educational Needs and Disabilities (SEND) policies.

Ethics and dissemination

Ethical approval was granted by the University of Bristol Faculty of Health Sciences Ethics Committee. Findings will contribute to the development of a full-scale cluster RCT to assess the effectiveness of the intervention with adequate statistical power. The results will also support the refinement of the intervention, and its underlying theory.

Keywords: Cerebral Visual Impairment, Complex intervention, feasibility trial, process evaluation, child.

Strengths and limitations of this study

- **Intervention developed with input from professionals and families**
- **Novel intervention to improve awareness of CVI and strategies to help**
- **Process evaluation designed to inform future full-scale trial, if warranted**
- **Study designed using programme logic to inform data collection timelines and methods**
- **Ethical approval re-established following COVD-19 related pause in the study**

For peer review only

INTRODUCTION

Damage or poor function in the brain areas related to vision leads to brain-related visual impairments, also known collectively as cerebral visual impairment (CVI) (1, 2). CVI in children can manifest in many ways; for example, being unable to find things in a cluttered page; bumping into people; being unable to copy from the class whiteboard to their own workbooks or difficulties controlling their eye position effectively to keep focused on a task (3). It has been reported that vision problems may be misinterpreted by teachers as behavioural, learning or intellectual difficulties (4) and so children with CVI may not get the help they need. Concern has been raised that missing a diagnosis of CVI can affect mental health(5, 6), and research from a UK birth cohort suggested that children with CVI may experience academic difficulties(7). Research into strategies to support children with CVI has suggested that providing a less cluttered visual environment may help (8, 9).

In preparation for this study, discussions with two parent advisory groups, and with an advisory group comprising local health and education professionals, have reinforced accounts that simple adaptations of the child's visual world can be very helpful. They emphasize children's improved sense of well-being when they can achieve more and are not chastised for behaviour resulting from their CVI. They have told us that access to help is uneven between and within different areas of the country, and therefore is inequitable, and that few teachers, SENCo (Special Educational Needs Co-ordinator) or Learning Support Assistants (LSAs) know about CVI. In general, only children with reduced visual acuity have access to specialist vision support teachers (QTVIs) and so many children with CVI but good visual acuity, are ineligible for their help. A prevalence study conducted by our group has indicated that there are likely to be at least 3% of children with CVI-related vision problems in primary schools in England and that these are largely but not exclusively within the subset of children already identified as needing extra educational help(10).

Aim of the process evaluation

The aim of the CVI Project process evaluation is to understand both the functioning of the trial processes and the intervention itself. This understanding is crucial to the development and decision to proceed to the full trial.

METHODS AND ANALYSIS

Patient and Public Involvement

We have spoken with two groups of parents whose children have special educational needs and/or CVI. They have been consulted at every stage of the research so far and provided guidance on which measures to use with parents, and which topics to include in qualitative interviews. During the study design phase, the parents advised us on the need to raise awareness in schools about CVI, and we changed our draft intervention pack to include specific resources to address this. They were clear that teachers should be reimbursed for their time to take part in the study and that we should outline the potential benefits of identifying CVI whereby the right strategies can be put in place, freeing up teacher time not spent trying ineffective interventions. They also advised us that schools might appreciate the benefit of being able to demonstrate to OFSTED (Office for Standards in Education) inspectors that they were implementing strategies to support students who may be struggling with their learning.

For peer review only

The CVI Project Intervention

We have developed an intervention pack which seeks to share knowledge with teachers so that they can make both universal and targeted changes within their school, to better support children with CVI and potentially other children too(9).

The development of the intervention pack is based on recent guidance, including a review of the published literature, using an iterative process of development and refining, involving key stakeholders, developing an underlying theory that takes into account the context and possible real world implementation issues (11). The key elements of the intervention and the hypothesised outcomes are summarised in a Logic model - see Figure 1.

We hypothesize that this multi-faceted approach involving both schools and the hospital service, will improve educational and wellbeing-related outcomes for any child with CVI and potentially for their peers as well. We have therefore devised an information-sharing pack to give schools this information. This includes an overview and explanations of the condition and gives suggestions as to how to make the school more "vision friendly". Central to this is to use universal approaches wherever possible - for instance decluttering walls and classrooms, using larger font and clear layouts with worksheets. Additional targeted strategies, for use with at-risk children, will also be given, with explanations about who might benefit from these, how they are intended to work and their potential impact. The information included in the intervention pack for teachers is collated from several centres of excellence that have developed materials about CVI, including Bartiméus in the Netherlands(12) and the international TeachCVI initiative.(www.Teachcvi,net) (13)The intervention also includes sharing information with the local paediatric ophthalmology clinics about the presentations of and support for children with CVI, so that they are happy to accept referrals of at-risk children. Intervention schools will be able to refer a maximum 5% of children to the paediatric ophthalmology clinic for a functional visual CVI assessment. A printout of the CVI assessment will be available to the family and school with strategies where applicable.

A logic model showing proposed mechanisms of change, impact and outcomes is shown in Figure 1. This model has been refined throughout our intervention development with parents and professionals, and includes consideration of unintended negative consequences of the intervention, or 'dark logic', as proposed by Bonell and colleagues (14).

Figure 1: CVI Project Intervention Logic Model

The CVI Project Feasibility Trial

The intervention and trial processes are being tested in a cluster randomised feasibility trial, with nested process and health economic evaluations. The main aim of this trial is to assess the feasibility and acceptability of our intervention and trial methods, to inform the development of a future adequately-powered cluster-randomised controlled trial, if warranted (15). The full protocol for the feasibility trial is presented separately and is summarised here, to give context for the process evaluation, which is described in full below.

The feasibility trial involves mainstream primary schools in three regions of the UK: Gloucester, Hampshire and Somerset. Baseline and follow up data will be collected on students in years 3-5 attending participating schools.

For peer review only - http://bmjopen.bmj.com/site/about/guidelines.xhtml

In designing the study, we were aware of 2 challenges; firstly, that in real life it would be impractical to examine all children to look for CVI and secondly that even if that were possible, once identified we would need to help the affected children and would not therefore have a control group. However, in our recent study, (10) we found that most children with CVI were found within the group of children getting extra help at school, and these children are easily identifiable by schools. In this feasibility trial therefore, the primary outcome (child self-reported mental wellbeing) will be assessed in all children who are getting extra help, rather than only in children with confirmed CVI. We will not assess every child in the study for CVI. (10) In addition, we also found some children with CVI-related vision problems who were not getting extra help and we hypothesize many children might benefit from some aspects of the intervention e.g.decluttering the classroom to make the visual environment less busy. We will therefore collect data for *all* children in years 3-5. The primary outcome of child-reported quality of life will be assessed in the children getting extra help, and we will include all children in an additional (secondary) analysis using the same outcome.

After baseline data are collected, a statistician separate from the study will randomise the participating schools, with two stratification variables to maintain similar sample sizes in each arm (16) 1) 15% or less of children having extra help or more than 15% of children and 2) size of school (3+ classes per year vs 2 or fewer classes per year, in years 3-5). Schools will be told immediately whether they are allocated to the control group (and will be asked to carry on as normal for the school year) or the intervention group (where they will be invited to implement the CVI intervention pack, described above).

Some of the outcomes of the feasibility study will be assessed using data collected during the study in a process evaluation. The process evaluation will use a mixture of quantitative and qualitative methods for data collection. Complex interventions are described as those which contain several interacting components and may also relate to the implementation of the intervention and its interaction with its context. (17) Process evaluations embedded within complex interventions aim to understand the functioning of an intervention by examining implementation, mechanisms of impact and contextual factors. (18) There are several frameworks which combine these aspects of process evaluation within feasibility of complex interventions and RCTs; for this study our framework is based on guidance from the Medical Research Council (MRC). (17) We have used this guidance to develop the aims and conceptual framework for this Process Evaluation (PE). These are described below.

The seven domains to be evaluated as recommended by MRC guidance are as follows:

1. *Implementation of the intervention*

This domain aims to discover what is implemented in practice. We will consider how the CVI Project Intervention Training package is used in each school. We have made suggestions for how schools may wish to implement the intervention (including delivering the presentation at a staff meeting and sharing the resources with class teachers), however we have not been prescriptive about this. Early discussions with teachers in our advisory groups suggested that individual schools would prefer to find their own ways to implement the intervention pack, and that autonomy over this process would be appreciated. We will assess the quality (fidelity) of the intervention and how much of the intervention (dose) was delivered and to whom (reach). We will also determine if there have been any adaptations to the intervention and the reason or purpose of these adaptations. Consideration will also be given to the core components of the intervention, and the barriers and facilitators which influence how individual components of the intervention are selected and delivered.

For peer review only - http://bmjopen.bmj.com/site/about/guidelines.xhtml

2. Mechanism of Impact/change

The process evaluation will consider how the intervention as delivered by schools, worked. Research questions will provide information and insights about how the participants, schools and parents responded to and interacted with the intervention. Questions will examine changes in the understanding and knowledge of staff about CVI and how this interacted with their implementation of the intervention, along with exploring the barriers and facilitators they experienced to implementation. Questions will also consider if there were any unintended or unexpected consequences or pathways of the intervention.

3. Programme Differentiation and Usual Practice

The research questions in this domain will examine how the intervention differed from usual practice in the intervention schools. Contamination from intervention to control schools is a potential risk, where schools in the control group make changes related to CVI after hearing about the intervention from a school in the intervention arm. We will seek to assess this with exploratory questions about how and why this occurred and the outcome for control schools.

4. Acceptability of the intervention

This domain will consider the interactions of participants, both school staff and parents with the intervention. The effects of acceptability on implementation of the intervention will be explored. We will consider if acceptability changes across the school year, and how the responses and interactions of staff and parents with the intervention might change the way the intervention is implemented.

5. Sustainability

This domain will consider whether the intervention could become part of normal practice after the feasibility trial was complete. We will assess the fidelity, dose and reach of the intervention in is sustained form. Where it has not been sustained, or there are no plans to continue with the changes after the trial, we will explore why this is. Barriers and facilitators to sustainability will also be explored.

6. Context

Contextual factors may influence the effectiveness of an intervention both directly and indirectly. These are external factors which can impede or strengthen its implementation or effects (MRC). We will consider how the paediatric ophthalmology clinics respond and interact with the intervention, including the referral pathway from schools to HES. In addition, this domain will consider the wider contextual factors which could affect the implementation of the intervention.

7. Trial Process

Understanding the participants responses to and interactions with the trial processes will allow an assessment of how the trial methods might require adjustment or re-design for further work. Understanding the responses of the participants will also provide information about recruitment and continued participation in the trial by both schools, teachers and parents.

Design

The CVI Project is a feasibility cluster randomised study with nested process evaluation and health economic assessment. The process evaluation will use both quantitative and qualitative data sources in order to answer our research questions. We anticipate that all schools will contribute data to the process evaluation, allowing for different experiences with the study between intervention and control arms.

For peer review only

Data sources

We have summarised the research domains, research questions and methods of evaluation in Table 1.

For peer review only

Table 1: Process evaluation research domains and methods of evaluation

Domain	Research questions<br><br>***Process evaluation framework mapping***	Informant/source of data	Methods	Timing of data collection	Analysis
**Implementation of intervention (the information pack)**<br><br>**Intervention Schools n=4**	Which CVI Project information pack components were delivered?<br><br>*Fidelity, dose, reach*	SENCO/head teacher<br>n=4	Brief survey question	Post-intervention	Data recorded and tabulated
	Which resources were used?<br><br>How were these resources used?<br><br>*Fidelity*	SENCO/head teacher<br>n=4	Brief survey questions<br><br>Semi-structured interview	Post-intervention	Data recorded and tabulated<br><br>Thematic analysis
	How was the training delivered? For example: training day, short sessions.<br><br>*Fidelity, adaptation*	SENCO/head teacher<br>n=4	Brief survey questions	Post-intervention	Data recorded and tabulated
	Who received the training?<br><br>*Reach*	SENCO/head teacher<br>n=4	Brief survey questions	Post-intervention	Data recorded and tabulated
	Were adaptations made to resources? How did this affect implementation?<br><br>*Adaptation, Fidelity*	SENCO/head teacher<br>n=4	Semi-structured Interview	Post-intervention	Thematic analysis
	What were the barriers and facilitators to delivering the training?<br><br>*Mediators*	SENCO/head teacher<br>n=4	Semi-structured Interview	Post-intervention	Thematic analysis
**Mechanisms of change of CVI Project intervention**<br><br>**Intervention Schools**	What was the response of staff to the training?<br><br>*Participant responses to and interactions with the CVI Project Intervention*	SENCOs/head teacher classroom teachers, LSA years 3-5<br>n=TBC	Semi-structured Interview	Post-intervention	Thematic Analysis

For peer review only

**n=4**	How did staff knowledge about CVI change in response to training/intervention?<br><br>*Participant responses, reach*	SENCO/head teacher classroom teachers,<br>LSAs years 3-5<br>n=TBC	Bartiméus Centre (Netherlands)<br>Teacher CVI Knowledge Assessment Survey	Brief online survey pre randomisation follow up data collection.	Quantitative analysis
	What were staff and parents' responses to the implementation of the CVI Project intervention?<br><br>*Participant responses to and interactions with the CVI Project Intervention*	SENCO/head teacher classroom teachers, LSA years 3-5<br>n=TBC	Semi-structured Interview	Post- intervention	Thematic analysis
	What changes to school environment took place? E.g. Whole school/class/individual child changes<br><br>*Fidelity, adaptations, and reach*<br>*Context of implementation of intervention*	SENCO/head teacher, classroom teachers, years 3-5<br>n=23 classrooms<br><br>SENCO/head teacher n=4	Photographs taken of study designated viewpoints of each wall in years 3-5 classrooms<br><br>Semi-structured interviews	Pre and post randomisation<br>Pre-follow up data collection<br><br>Post follow up data collection	Quantitative analysis using validated University of Bristol 'clutter' measure<br><br>Thematic analysis
	Were there any unintended/unexpected consequences from the intervention? What were they? How could they be mitigated (if necessary)?<br><br>*Unintended/unexpected pathways or consequences*	SENCO/head teacher, classroom teachers, LSAs years 3-5; parents<br>n=TBC	Semi-structured Interview	Post-intervention	Thematic analysis
	What were the barriers and facilitators for the implementation of the CVI Project intervention?<br><br>*Participant responses to and interactions with the CVI Project Intervention*	SENCO/head teacher, classroom teachers, LSAs years 3-5; parents<br>n=TBC	Semi-structured Interview	Post-intervention	Thematic analysis
**CVI Project intervention differentiation and usual practice**	Was any contamination detected from intervention to control schools?	SENCO/head teacher<br>n=7 (all schools)<br><br>Head teacher/SENCO and classroom teachers, years 3-5	Semi-structured Interview<br><br>Photographs of study designated viewpoints of	Follow up data collection	Thematic analysis

For peer review only

**Intervention schools n=4**<br><br>**Control Schools n=3**	Were there any changes in school SEND documentation over the course of the study that might indicate contamination?<br><br>*Contamination*	n=35 classes (control and intervention schools)<br><br>SEND Policy documents in all schools<br>n=7	each wall in years 3-5 classrooms<br><br>Structured review of schools' SEND documentation	Pre-randomisation and at follow up data collection<br><br>Pre-follow up data collection	Validated University of Bristol 'clutter' measure<br>Quantitative analysis<br><br>Content analysis
	What was different about the CVI Project Intervention when compared to usual practice within the school?<br><br>*Differentiation, mediators to implementation of the intervention*	Head teacher/SENCO and classroom teachers, years 3-5<br>n=TBC	Semi-structured Interview	Follow up data collection	Thematic analysis
**Acceptability of the Intervention**	Was the CVI project intervention acceptable to school staff and parents?<br><br>*Acceptability: participants' responses and interactions with the intervention*	Intervention schools: SENCO/head teacher, classroom teacher/LSA years 3-5, parents<br>n=TBC	Semi-structured Interview	Post Follow-up	Thematic analysis
	What were the experiences of staff and parents of taking part in the CVI project intervention?<br><br>*Participant interactions with the CVI Project Intervention*	Intervention schools: SENCO/head teacher, classroom teachers, LSAs years 3-5; parents<br>n=TBC	Semi-structured Interview	Post Follow-up	Thematic analysis
**Sustainability of the intervention**	Did schools/were schools intending to continue with intervention after the trial period?<br><br>*Sustainability, participants' responses to the intervention, fidelity, quality of sustained use.*	SENCO/head teacher<br><br>n=4	Semi-structured Interview	Post Follow-up	Thematic analysis
	How was the intervention integrated into usual practice?<br><br>Was intervention integrated into SEND policies?	SENCO/head teachers, classroom teachers, years 3-5<br>n=TBC<br><br>SEND policy documents from intervention schools n=4	Semi-structured Interview<br><br>Structured review of SEND policy documents	Post Follow-up<br><br>Pre-randomisation<br>Post Follow-up	Thematic analysis<br><br>Content analysis

For peer review only

	Mechanisms of sustainability of intervention				
	What factors made it more/less likely to be sustained/adopted into usual practice?<br><br>*Barriers and facilitators/mediators to sustainability of the intervention*	SENCO/head teacher<br><br>n=4	Semi-structured Interview	Post Follow-up	Thematic analysis
**Context**<br><br>**Intervention Schools n=4**	What were the experiences of paediatric ophthalmology clinics and orthoptists?<br><br>*Participants responses to the intervention*<br>*Barriers and facilitators to participation in the CVI Project feasibility RCT*	Head orthoptist<br>n=3	Semi-structured Interview	Post Follow-up	Thematic analysis
	How did the referral process from schools to paediatric ophthalmology clinics work?<br><br>*Fidelity, adaptations, reach, barriers and facilitators*<br>*Contamination, unintended consequences*	Paediatric ophthalmology clinics<br>n=3<br>SENCO/head teacher<br>n=4	Semi-structured Interview	Post Follow-up	Thematic analysis
	How many children were given study referral letters for their parents?<br>Who were they referred to?<br><br>What were the characteristics of the children who attended the paediatric ophthalmology clinic with a study referral letter?<br><br>*Fidelity, dose, reach*	SENCO/head teacher<br>n=4<br><br>Paediatric ophthalmology clinics<br>n=3	Records kept at each school<br>Schools collate data<br><br>CVI-vision assessment Conducted by orthoptists	Throughout trial	Count and record<br><br>Recorded on University REDCap database accessed via paediatric ophthalmology clinics.
	How did contextual factors affect intervention implementation? (e.g. funding pressures; reorganisation; academisation, Ofsted, SATS)<br><br>*Implementation and mechanisms of impact*	SENCO/head teacher<br>n=4	Semi-structured Interview	Pre-follow up data collection	Thematic analysis

**Trial Process**<br><br>**Intervention and Control Schools n=7**	What did schools think about the recruitment process?<br><br>*Trial methods, acceptability to participants. Participant responses to trial methods Barriers and facilitators*	Head teachers/SENCOs in control and intervention schools<br><br>n=7	Semi-structured Interview	Post baseline data collection	Thematic analysis
	What was the initial recruitment rate?<br><br>*Recruitment data*	Demographic data from publicly available government schools data base	Extraction of demographic data	Post recruitment	Descriptive data summaries
	What was the dropout rate of schools from the research? What reasons were given?<br><br>*Participant responses and interactions with the trial processes*	Recorded as part of trial routine data collection	Quantitative data with some free text/coded responses for reasons	Ongoing throughout study period	Count and record
	How many parents withdrew their children from the study? What reasons were given?<br><br>*Participant responses and interactions with the trial processes*	Recorded as part of trial routine data collection	Quantitative data with some free text/coded responses for reasons	Ongoing throughout study period	Count and record
	How did schools communicate with parents about trial?<br>*Mechanisms of impact, fidelity, reach*	Examination of school documents/websites/newsletters	Structured review of documents	Ongoing throughout study period	Content analysis
	Acceptability of trial processes: how did schools respond to randomisation? Including the arm of the trial that they were allocated to?<br><br>*Acceptability of trial processes, interactions of participants with trial processes*	SENCO/head teacher in intervention and control schools<br><br>n=7	Semi-structured interview	Post randomisation	Thematic analysis

For peer review only

Surveys
Each intervention school will complete a brief school-level survey during the intervention period, conducted at the beginning of the qualitative interview. The survey includes 6 questions about the implementation of the intervention pack including how it was delivered, who delivered it, which components of the intervention pack have been used, who received the training, how many referral letters have been sent out and how many students have been identified for referral. Follow up questions will include numbers who have received the intervention, which components have been used, numbers of children identified and referred.

Teacher knowledge of CVI will be assessed before randomisation and at follow up, using the Bartiméus Centre (Netherlands) Teacher CVI Knowledge Assessment Survey (12). All teachers for years 3-5 in all schools will complete these. The survey is comprised of 5 statements about knowledge of CVI (e.g. 'I understand the impact of CVI in daily life') and respondents are asked to rate whether they feel each statement is true for them on a scale of 0-10.

Qualitative interviews with school representative (all schools)
One key representative from each school (usually the SENCO or head teacher) will take part in a semi-structured interview at baseline following randomisation, (after the intervention has been delivered in intervention schools) and at follow up. The initial interview will cover school ethos, experiences with data collection, trial processes (including randomisation) and any changes during the study period. Additional topics for intervention schools will include responses to the intervention pack, resources implemented, feedback from staff, monitoring and recording of changes. See Supplementary materials for topic guides.

Qualitative interviews with school staff (intervention schools only)
School staff will be invited to take part in a semi-structured interview post intervention implementation, to share their experiences of the CVI Project intervention. The interviews will explore reactions to the intervention materials, how they have been used, views and opinions on their use and usefulness, changes to provision, implementation of both universal and targeted strategies and any feedback on the data collection methods used in the trial. Interviews with staff will take place as the intervention is being used at the school.

Qualitative interviews with parents (all schools)
Parents who returned 'consent to contact' forms will be contacted and invited to take part in a semi-structured interview about their experiences with the study. Interviews will explore their experiences with the data collection, surveys and any thoughts they had about randomisation. Interviews will also cover any changes during the study period and what these can be attributed to, and any referral processes and experiences with the paediatric ophthalmology clinics. Any positive, negative or unintended consequences will be discussed.

Qualitative interviews with paediatric ophthalmology clinic staff
Paediatric ophthalmology clinic staff with involvement in the study will be invited to take part in a semi-structured interview to explore their experiences with referrals from schools. The topics covered in these interviews will include the referral process, characteristics of children who are referred, communication between school, family and hospital unit.

Classroom Clutter (all schools)

For peer review only

We will ask schools to take photographs of each wall in the classrooms used by years 3-5 in each school, pre-randomisation and again at follow up. Photographs of the classrooms at the start and end of the study period will be compared and scored subjectively for the amount of clutter, by observers unaware of the school or time they were taken. We will also pilot using an adaptation of a visual clutter quantification system originally used in a study of how the visual characteristics of a natural scene affect bird and human search behaviour(19). We anticipate that we will present the results of analyses using the photographs briefly in our results paper and separately in full.

School document analysis

We will investigate whether there any changes in school SEND documentation over the course of the study that might indicate contamination by conducting a structured review of all school's SEND policy documentation pre-randomisation and at follow up. They will be studied for any changes made during the study period. Changes found will be included as topics for discussion with SENCo and staff interviews.

Analysis of qualitative data

With consent, the semi-structured interviews will be audio recorded and then transcribed verbatim, anonymised, checked for accuracy and imported into NVivo12(20). Thematic analysis using a continuous comparative method will be used to identify recurrent ideas and patterns within the data (21). Transcripts will be independently coded by two researchers to enhance rigour by cross checking the coding strategy and early development of themes. During analysis, themes will be discussed among the team until a final consensus on the nature and phrasing of key themes and subthemes is reached. Anonymised quotes will be presented to illustrate the main themes.

Analysis of quantitative data

Quantitative survey data will be presented as counts and percentages, as appropriate. String variables will be coded into categories. The CVI knowledge survey will be scored and comparisons made between baseline and follow up. Survey data will be compared between intervention and control schools, but this will be exploratory and will be reported descriptively as the study is not powered to detect an effect size (15).

Photographs of the classroom setting will be analysed using a clutter rating scale, developed at baseline with independent blind ratings by an academic not connected with the study, but with expertise in this area.

School policy documents will be subject to content analysis by the study team, using a data extraction table to identify any differences between schools at the beginning and end of the study period.

ETHICS and HRA

Ethical approval for the study was obtained from the University of Bristol Faculty of Health Sciences Ethics Committee (FREC Ref: 89144). The study is registered on the International Standard Randomised Controlled Trial Number registry (registration number: ISRCTN13762177). Approval from the Health Research Authority (HRA- Ref 19/HRA/6124) has been obtained.

Full details of consent procedures are described in our feasibility trial protocol paper (in preparation), but in brief: Head teachers agree to participate on behalf of the children and staff in his/her school, information sheets will be provided to school staff and parents, parents will be able to withdraw their child's data by completing a non-agreement form. Verbal agreement for child

For peer review only

completed measures will also be sought and questionnaires will not be completed by any child who does not wish to do so. A link to the full Privacy notice online (22)will be provided to all parents and school staff.

Conclusions

We have presented a protocol for a detailed process evaluation of a complex information sharing intervention to improve the mental well-being of primary school children with CVI. Development of an intervention to support children with CVI has been requested by parents (23), and we have developed it with their input. This feasibility study will assess whether and how a future trial should establish effectiveness of the intervention with feedback from this process evaluation. We have addressed key domains as per MRC guidance, and have developed our process evaluation research questions around these aims. Data gathered will help us to interpret the findings from the trial, but importantly, will help to refine the design, content and delivery of the intervention. We have also included data to improve trial processes and communication between schools and the research team in a future full-scale trial. Given that this is a process evaluation for a feasibility trial, we will not be powered to interpret the primary outcome differences between intervention and control, and the data gathered as part of the process evaluation will be limited in explaining any differences in the main feasibility study outcomes.

TRIAL STATUS

Favourable opinion by the University of Bristol faculty ethics committee was obtained on 12th August 2019 (Ref: 89144). We completed recruitment and baseline data collection from participating schools in all areas. We have recruited 7 schools to the feasibility trial and completed baseline trial data collection in February 2020. Four schools have been allocated to the intervention arm and three to control. Due to the COVID-19 global pandemic and school closures, we paused the study from March - September 2020. We have since obtained ethical approval for minor amendments relating to adjustments for a 'COVID-secure' restart to the study on September 1st 2020, with an extension to the study period until April 30th 2021. We will describe all COVID related changes to the study in our final results paper, as these relate to the findings of our feasibility study.

FUNDING AND SPONSORSHIP

University of Bristol provides Sponsorship for this study. The study is funded by the National Institute for Health Research (NIHR) Senior Research Fellowship ref: SRF-2015-08-005. The views expressed in this publication are those of the authors and not necessarily those of the NIHR, NHS or the UK Department of Health and Social Care.

Author contributions

CW led the development of the research including the design of the intervention and trial methods, with support from AP, TG and PS. RW provided data management and systems support. PS provided governance support. AP and TG led on the design of the process evaluation and associated research methodology. KB led the development of the health economics aspect of the process evaluation. All authors contributed to the final manuscript providing comments and text for the final submitted draft.

For peer review only

Acknowledgements

We are grateful to Ms Mai Baquedano for her support with online consent and questionnaires, via the REDCap system. We are also grateful to our family and professionals advisory group members who provided valuable insight into recruitment and data collection strategies, and input into the intervention delivery.

Competing interests

All the authors declare they have no competing interests

REFERENCES

1. Sakki HEA, Dale NJ, Sargent J, Perez-Roche T, Bowman R. Is there consensus in defining childhood cerebral visual impairment? A systematic review of terminology and definitions. The British journal of ophthalmology. 2018;102(4):424-32.
2. Dutton GN. Cognitive vision, its disorders and differential diagnosis in adults and children: knowing where and what things are. Eye (Lond). 2003;17(3):289-304.
3. Dutton GN, Jacobson LK. Cerebral visual impairment in children. Seminars in neonatology : SN. 2001;6(6):477-85.
4. Swift S, Davidson R, Weems L. Cortical Visual Impairment: Presentation Intervention and Prognosis in Educational Settings. TEACHING Exceptional Children PLus. 2008;4(5):1-14.
5. Lueck A, Dutton G. Vision and the Brain: AFB Press; 2015.
6. Murphy M, Fonagy P. Chief Medical Officers Report 2012. Chapter 10. Mental health problems in children and young people. In: Health UgDo, editor.: UK Government; 2012.
7. Williams C, Northstone K, Sabates R, Feinstein L, Emond A, Dutton GN. Visual perceptual difficulties and under-achievement at school in a large community-based sample of children. PLoS One. 2011;6(3):e14772.
8. McDowell N, Budd B J VI and Blindness. The perspectives of teachers and paraeducators on the relationship between classroom clutter and learning experiences for students with cerebral visual impairment. 2018;112(3):248-60.
9. Fisher AV, Godwin KE, Seltman H. Visual environment, attention allocation, and learning in young children: when too much of a good thing may be bad. Psychol Sci. 2014;25(7):1362-70.
10. Williams C, Pease A, Warnes P, Harrison S, Pilon F, Hyvarinen J, et al. Cerebral visual impairment (CVI)-related vision problems in children attending primary schools in England: Cross-sectional survey with nested case-finding study. Developmental Medicine & Child Neurology. 2020;in press.
11. O'Cathain A, Croot L, Duncan E, Rousseau N, Sworn K, Turner KM, et al. Guidance on how to develop complex interventions to improve health and healthcare. 2019;9(8):e029954.
12. Pilon-Kamsteeg F, Dekker-Pap MJ, de Wit GC, van Genderen MMJBJoVI. CVI Experience toolbox: Simulation of visual processing difficulties. 2019;37(3):248-57.
13. B&H The National Institute for the Blind Visually Impaired and Deafblind in Iceland. TeachCVI [Available from: https://www.teachcvi.net/.
14. Bonell C, Jamal F, Melendez-Torres G, Cummins SJJECH. 'Dark logic': theorising the harmful consequences of public health interventions. 2015;69(1):95-8.

For peer review only

15. Eldridge SM, Lancaster GA, Campbell MJ, Thabane L, Hopewell S, Coleman CL, et al. Defining feasibility and pilot studies in preparation for randomised controlled trials: development of a conceptual framework. PloS one. 2016;11(3):e0150205.
16. Treasure T, MacRae KD. Minimisation: the platinum standard for trials?: randomisation doesn't guarantee similarity of groups; minimisation does. British Medical Journal Publishing Group; 1998.
17. Moore G AS, Barker M, Bond L, Bonell C, Hardeman W, Moore L, O'Cathain A, Tinati T, Wight D, Baird J. Process evaluation of complex interventions: Medical Research Council guidance. MRC Population Health Science Research Network, London, 2014 Process evaluation of complex interventions: Medical Research Council guidance. London: MRC Population Health Science Research Network; 2014.
18. Grant A, Treweek S, Dreischulte T, Foy R, Guthrie BJT. Process evaluations for cluster-randomised trials of complex interventions: a proposed framework for design and reporting. 2013;14(1):15.
19. Xiao F, Cuthill IC. Background complexity and the detectability of camouflaged targets by birds and humans. Proceedings of the Royal Society B: Biological Sciences. 2016;283(1838):20161527.
20. Ltd. QIP. NVivo (Version 12) 2018 [Available from: https://www.qsrinternational.com/nvivo-qualitative-data-analysis-software/home.
21. Braun V, Clarke V. Thematic analysis. 2012.
22. The CVI Project Feasibility Study Data Security and Privacy Notice [Available from: http://www.thecviproject.co.uk/privacy/.
23. Jackel B, Wilson M, Hartmann EJJoVI, Blindness. A survey of parents of children with cortical or cerebral visual impairment. 2010;104(10):613-23.

For peer review only

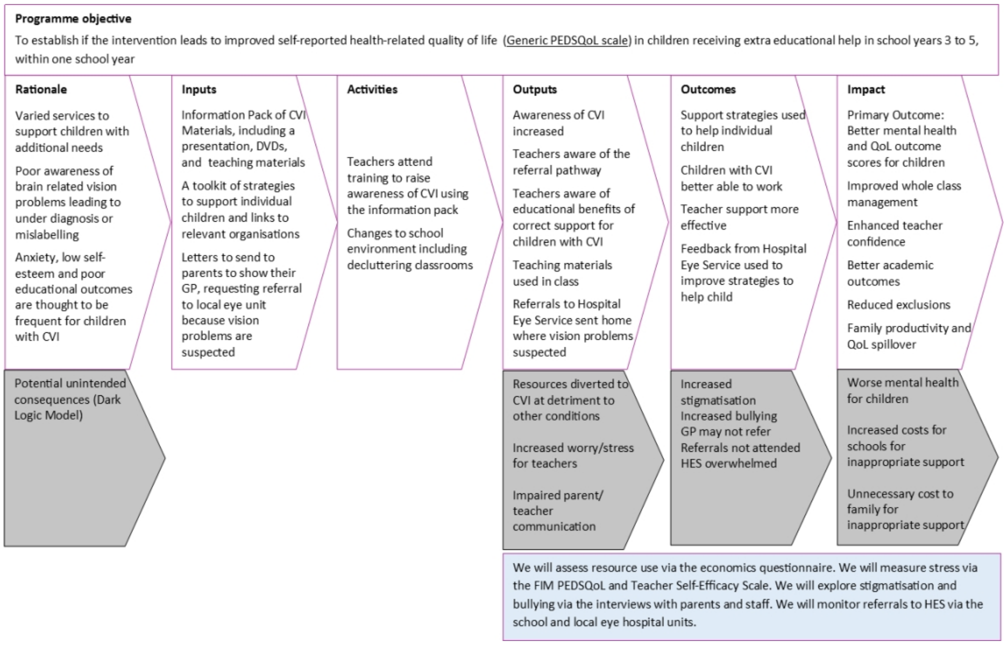
Programme objective
To establish if the intervention leads to improved self-reported health-related quality of life (Generic PEDSQoL scale) in children receiving extra educational help in school years 3 to 5, within one school year
Rationale
Varied services to support children with additional needs
Poor awareness of brain related vision problems leading to under diagnosis or mislabelling
Anxiety, low self-esteem and poor educational outcomes are thought to be frequent for children with CVI
Inputs
Information Pack of CVI Materials, including a presentation, DVDs, and teaching materials
A toolkit of strategies to support individual children and links to relevant organisations
Letters to send to parents to show their GP, requesting referral to local eye unit because vision problems are suspected
Activities
Teachers attend training to raise awareness of CVI using the information pack
Changes to school environment including decluttering classrooms
Outputs
Awareness of CVI increased
Teachers aware of the referral pathway
Teachers aware of educational benefits of correct support for children with CVI
Teaching materials used in class
Referrals to Hospital Eye Service sent home where vision problems suspected
Outcomes
Support strategies used to help individual children
Children with CVI better able to work
Teacher support more effective
Feedback from Hospital Eye Service used to improve strategies to help child
Impact
Primary Outcome: Better mental health and QoL outcome scores for children
Improved whole class management
Enhanced teacher confidence
Better academic outcomes
Reduced exclusions
Family productivity and QoL spillover
Potential unintended consequences (Dark Logic Model)
Resources diverted to CVI at detriment to other conditions
Increased worry/stress for teachers
Impaired parent/ teacher communication
Increased stigmatisation
Increased bullying
GP may not refer
Referrals not attended
HES overwhelmed
Worse mental health for children
Increased costs for schools for inappropriate support
Unnecessary cost to family for inappropriate support
We will assess resource use via the economics questionnaire. We will measure stress via the FIM PEDSQoL and Teacher Self-Efficacy Scale. We will explore stigmatisation and bullying via the interviews with parents and staff. We will monitor referrals to HES via the school and local eye hospital units.

Supplementary materials

A. Teacher CVI knowledge assessment survey

	0	1	2	3	4	5	6	7	8	9	10
1. I know everything about CVI	Not at all										Very much so
2. I understand how complicated the cerebral visual system is	Not at all										Very much so
3. I understand the impact of CVI in daily life	Not at all										Very much so
4. I can explain the impact of CVI in daily life to others.	Not at all										Very much so
5. I know how to adjust the environment of a child with CVI	Not at all										Very much so

B. School staff Interview Topic Guides (Control and Intervention)

CONTROL SCHOOLS TOPIC GUIDE

Introduction

- Check participant is still happy to be involved with the research
- Explain recording device and confidentiality
- Explain purpose of the interview and that participant does not have to talk about anything they feel uncomfortable with and can stop at any time for any reason

Your role
Topics to include:

- Years in role
- Involvement with SEN
- Brief School ethos overall

Experiences with the CVI Project survey collection
Topics/questions to include:

- What was it that first interested you in the study?
- How has communication been with the study team? Anything that we could have done better or made it easier for you (as a member of staff?)
- What did you think about the research agreement process?
- Data collection-how did you feel about that?
- Were the questionnaires? acceptable? Interesting? Too long? Too short?
- Randomisation experiences and feelings
- Classroom photographs

Changes during study period
Topics to include:

For peer review only

- Changes to provision, SEN or other
- How are changes monitored and recorded?
- Are they related to the study or not?
- Changes to policy documents -where did those changes come from? And how does that impact on your practice
- Positive, negative or unintended consequences of trial participation

INTERVENTION SCHOOLS TOPIC GUIDE

Introduction

- Check participant is still happy to be involved with the research
- Explain recording device and confidentiality
- Explain purpose of the interview and that participant does not have to talk about anything they feel uncomfortable with and can stop at any time for any reason

Your role

Topics to include:

- Years in role
- Involvement with SEN
- Brief School ethos overall

Brief Survey Questions

1. **How was the training delivered?**
2. **Who delivered the training?**
3. **Which components of the intervention pack were used?**
4. **Who received the training? How many people in each group and in total?**
5. **How many referral letters sent out to parents if any so far?**
6. **How many students/children identified as would benefit form a referral so far?**

Experiences with the CVI Project survey collection

Topics/questions to include:

- What was it that first interested you in the study?
- How has communication been with the study team? Anything that we could have done better or made it easier for you (as a member of staff?)
- What did you think about the research agreement process?
- Data collection-how did you feel about that?
- Were the questionnaires? acceptable? Interesting? Too long? Too short?
- Randomisation experiences and feelings
- Classroom photographs

Changes during study period

Topics to include:

- **The intervention pack**: initial thoughts about the content and presentation?
- **Training provided**, scope, reach, whose responsibility was it?
- **The intervention**
  - Time and impact on teachers/school/yrs 3, 4 and 5 up to July 2020; yrs 4, 5 and 6 from September 2020
  - What was engagement like from staff?
  - Use in real life, which strategies were used and for who? How did that go?
  - Changes to provision, SEN or other
  - How were changes monitored and recorded?

For peer review only

- Referrals to eye service experiences, process, feedback, changes, would onsite assessments be better?
    - Changes to policy documents -where did those changes come from? Any intention to change policy in future following intervention?
- Positive, negative or unintended consequences of trial participation
- Accessing specialist provision post-covid, how and source of provision/support

C. Parent interview topic guide (Control and Intervention)

Introduction

- Check participant is still happy to be involved with the research
- Explain recording device and confidentiality
- Explain purpose of the interview and that participant does not have to talk about anything they feel uncomfortable with and can stop at any time for any reason

Your role

Topics to include:

- Describe your children
- Involvement with SEN – existing service use, referrals and stage of process.
- School ethos overall

Experiences with the CVI Project survey collection

Topics to include:

- Data collection: paper forms, returning process, any problems?
- Surveys - acceptable? Interesting? Too long? Too short?
- Randomisation experiences and feelings
- Anything not clear or troubling in surveys?

Interview part 2: Changes during study period

Topics to include:

- Control schools:
    - Changes to provision, SEN or other
    - Did you notice any changes in what is provided for your child at school?
    - Are they related to the study or not? eg classroom changes?
    - Did this impact on anything at home?
    - Positive, negative or unintended consequences of trial participation

- Intervention schools:
    - Changes to provision, SEN or other
    - Did you notice any changes in what is provided for your child at school?
    - Are they related to the study or not? eg classroom changes?
    - Did this impact on anything at home?
    - Positive, negative or unintended consequences of trial participation
    - Referrals to eye service experiences, process, feedback, changes, useful?

For peer review only

D. Hospital eye-service staff interview topic guide

Introduction

- Check participant is still happy to be involved with the research
- Explain recording device and confidentiality
- Explain purpose of the interview and that participant does not have to talk about anything they feel uncomfortable with and can stop at any time for any reason

Your role

Topics to include:

- Years in role
- Involvement with SEN
- Involvement with CVI –previous experience of managing children with CVI

Experiences with the CVI project referral process

Topics to include:

- How did the referral process from schools to eye unit work?
- Timing of referrals, accuracy, ability to book appointments
- Managing additional workload
- Scaling up of intervention if helpful - potential barriers/problems

Experiences with the CVI Project data collection

Topics to include:

- Training to do the visual assessments, experiences and opinions
- Patient expectations for referrals, problems identified, any diagnoses?
- Anything you would change in the research data collection (the Looking Activities)?
- Data entry using REDCap, easy to use?

Feedback to families and schools

Topics to include:

- Parent/carer reactions to appointments and feedback
- Any school responses or contact

Overall experience

Topics to include:

- Positive, negative or unintended consequences of trial participation

For peer review only
